# Re-analysis of Whole Genome Sequence Data From 279 Ancient Eurasians Reveals Substantial Ancestral Heterogeneity

**DOI:** 10.3389/fgene.2018.00268

**Published:** 2018-07-20

**Authors:** Daniel Shriner

**Affiliations:** Center for Research on Genomics and Global Health, National Human Genome Research Institute, Bethesda, MD, United States

**Keywords:** ancestry, ancient DNA, Bronze Age, gene flow, migration, Neolithic, population structure

## Abstract

Supervised clustering or projection analysis is a staple technique in population genetic analysis. The utility of this technique depends critically on the reference panel. The most commonly used reference panel in the analysis of ancient DNA to date is based on the Human Origins array. We previously described a larger reference panel that captures more ancestries on the global level. Here, I reanalyzed DNA data from 279 ancient Eurasians using our reference panel. I found substantially more ancestral heterogeneity than has been reported. Reanalysis provides evidence against a resurgence of Western hunter-gatherer ancestry in the Middle to Late Neolithic and evidence for a common ancestor of farmers characterized by Western Asian ancestry, a transition of the spread of agriculture from demic to cultural diffusion, at least two migrations between the Pontic-Caspian steppes and Bronze Age Europe, and a sub-Saharan African component in Natufians that localizes to present-day southern Ethiopia.

## Introduction

Before the technological advances that permitted ancient DNA studies, historical inferences were made using present-day samples in conjunction with well-established theory and techniques in population genetics and phylogenetic reconstruction. Inferences regarding population structure have been based on the popular software STRUCTURE (Pritchard et al., 2000), ADMIXTURE (Alexander et al., 2009), and variants thereof. The basic idea is to perform model-based estimation of ancestries from multi-locus genotypes. Having learned ancestry-specific allele frequencies in unsupervised clustering analysis from a data set, it is computationally efficient to project new samples onto the ancestries in order to learn about population structure in the new samples. The utility and quality of projection analysis, or supervised clustering analysis, strongly depends on the reference panel of learned ancestries.

Commercially available and widely used genotyping arrays designed for studies of human medical genetics have unknown patterns of marker ascertainment bias. This ascertainment bias reflects differences in the allele frequency spectra in non-European populations compared to European populations as well as enrichment of array content for ancestry informative markers and other content specifically of medical value. The Human Origins genotyping array is a collection of 13 panels, each designed with known ascertainment, for studies of human population genetics (Patterson et al., 2012). In the analysis of ancient DNA, the reference panel most widely used to date comprises <3,000 individuals genotyped using the Human Origins array (Lazaridis et al., 2014; Günther et al., 2015; Mathieson et al., 2015; Cassidy et al., 2016), although some data are not freely available.

One consequence of use of a single reference panel is consistency within the ancient DNA field. Unfortunately, the labels for ancestries used in these papers lacks overlap with labels used by other researchers for ancestries in present-day peoples. Furthermore, none of the results in the ancient DNA papers has been replicated using a second reference panel. We previously combined completely public domain data to generate a reference panel comprising 5,966 individuals from 282 samples, from which we estimated 21 ancestries (Baker et al., 2017). Our reference panel covers more ancestral diversity than the Human Origins-based panel, with no evidence of marker ascertainment bias (Baker et al., 2017). Using this reference panel, I investigated (1) the reproducibility of findings previously reported in ancient DNA studies and (2) the impact of broader coverage of ancestries. After projecting 279 ancient Eurasians onto our reference panel, I reached a distinct series of conclusions regarding the genetic history of Europe and Western Asia.

## Materials and methods

### Ancient DNA data

I retrieved and integrated data from 279 ancient Eurasians from 49 samples (Keller et al., 2012; Lazaridis et al., 2014, 2016; Skoglund et al., 2014; Allentoft et al., 2015; Günther et al., 2015; Jones et al., 2015; Mathieson et al., 2015; Broushaki et al., 2016; Hofmanová et al., 2016). Data were provided either as called genotypes in VCF files or aligned sequences in BAM files. To generate the most probable genotypes from aligned sequences, BAM files were processed using the program bam2mpg using a quality filter of 20 and the reference human genome sequence hs37d5 in fasta format (available at https://github.com/nhansen/bam2mpg), with results saved in VCF files. For each of the 279 individuals, sampling locations are shown in Figure S1 and meta-data is provided in Table S1.

### Supervised clustering

The global reference panel was previously described (Baker et al., 2017). Briefly, this panel consists of ancestry-specific allele frequencies for 21 ancestries and 19,075 SNPs, generated from unsupervised clustering of 5,966 individuals from 282 global samples. The present-day geographic distributions of the 21 ancestries are shown in Figure S2.

Genotype data were extracted from VCF files using VCFtools version 0.1.14 (Danecek et al., 2011). Supervised clustering was performed by projecting the ancient Eurasians onto the reference panel using ADMIXTURE version 1.3 (Alexander et al., 2009). Standard errors were obtained from 200 bootstrap replicates. Inverse variance weighting was used to combine ancestry proportions across individuals within samples, accounting for both within- and between-individual variance. Assuming approximate normality, sparsity was induced by zeroing out any ancestry for which the 95% confidence interval included 0. Finally, the significant ancestry proportions were renormalized to sum to 1.

### Ethics

This project was determined to be excluded from IRB Review by the National Institutes of Health Office of Human Subjects Research Protections, Protocol 17-NHGRI-00282.

## Results

### European hunter-gatherers

**Figure 1:**
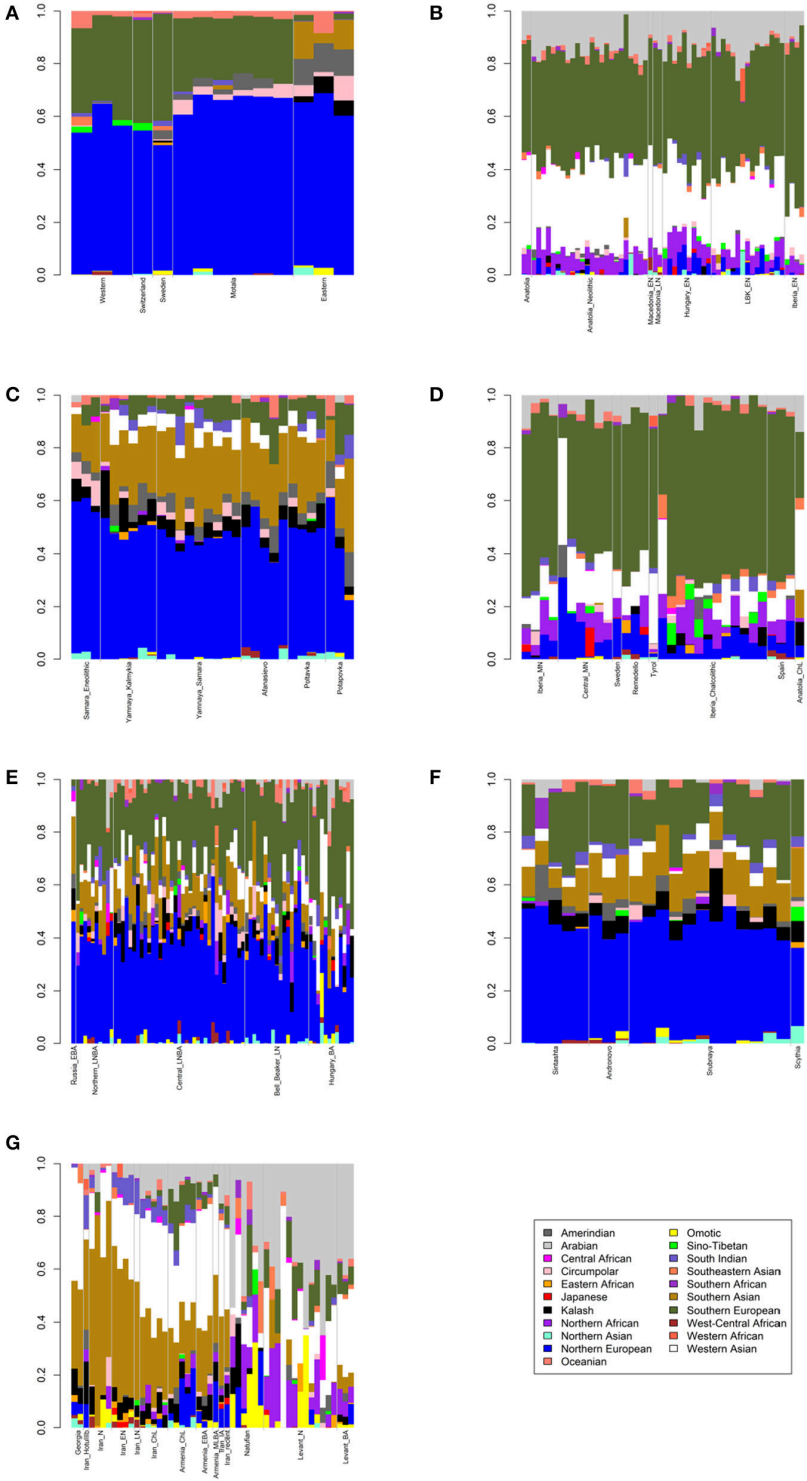
Admixture bar plots showing projections of ancient Eurasians onto 21 ancestries. The proportions are the raw output from ADMIXTURE. **(A)** European hunter-gatherers. **(B)** Early Neolithic peoples. **(C)** Eneolithic to Middle Bronze Age steppe peoples. **(D)** Middle Neolithic to Copper Age European peoples. **(E)** Bronze Age European peoples. **(F)** Late Bronze to Iron Age steppe peoples. **(G)** Western Asian peoples.

First, I considered hunter-gatherers from Hungary, Luxembourg, and Spain (Western), Switzerland, Sweden (Sweden and Motala), and Karelia and Samara (Eastern, Figure 1A). Overall, these 14 hunter-gatherers averaged 71.6% Northern European ancestry, 27.4% Southern European ancestry, and 0.9% Oceanian ancestry (Table 1). Y DNA haplogroups included eight I2, one C1, one J, one R1a, and one R1b (Table 2). Mitochondrial haplogroups included 12 U, one C, and one R (Table 2). Amerindian, Circumpolar, and Southern Asian ancestries decreased from east to west, complemented by an increase of Southern European ancestry (Table 1).

**Table 1:** Mean ancestry proportions for 49 samples of ancient Eurasians.

**Group**	**Sample**	**Size**	**Northern Asian**	**West-Central African**	**Omotic**	**Northern European**	**Japanese**	**Eastern African**	**Kalash**	**Northern African**	**Sino-Tibetan**	**Circumpolar**	**Amerindian**	**Southern Asian**	**Western Asian**	**Central African**	**Southeastern Asian**	**South Indian**	**Southern European**	**Southern African**	**Oceanian**	**Western African**	**Arabian**
European Hunter-gatherers	Western	3	0	0	0	0.601	0	0	0	0	0	0	0	0	0	0	0	0	0.372	0	0.027	0	0
	Switzerland	1	0	0	0	0.581	0	0	0	0	0	0	0	0	0	0	0	0	0.419	0	0	0	0
	Sweden	1	0	0	0	0.519	0	0	0	0	0	0	0.037	0	0	0	0	0	0.444	0	0	0	0
	Motala	6	0	0	0	0.673	0	0	0	0	0	0.037	0.021	0	0	0	0	0	0.244	0	0.025	0	0
	Eastern	3	0	0	0	0.662	0	0	0.048	0	0	0.066	0.109	0.115	0	0	0	0	0	0	0	0	0
Early Neolithic	Anatolia	2	0	0	0	0	0	0	0	0.091	0	0	0	0	0.355	0	0	0	0.437	0	0	0.011	0.106
	Anatolia_Neolithic	24	0	0	0	0	0	0	0	0.069	0	0	0	0	0.345	0	0	0	0.424	0	0.006	0	0.157
	Macedonia_EN	1	0	0.050	0	0	0	0	0	0.077	0	0	0	0	0.360	0	0	0	0.419	0	0	0	0.093
	Macedonia_LN	2	0	0	0	0	0	0	0	0.069	0	0	0	0	0.335	0	0	0	0.463	0	0	0	0.133
	Hungary_EN	10	0	0	0	0	0	0	0	0.085	0	0	0	0	0.273	0	0	0	0.486	0	0	0	0.156
	LBK_EN	15	0	0	0	0	0	0	0	0.055	0	0	0	0	0.319	0	0	0	0.492	0	0	0	0.134
	Iberia_EN	4	0	0	0	0	0	0	0	0.049	0	0	0	0	0.194	0	0	0	0.657	0	0	0	0.100
Eneolithic to Middle Bronze Age Steppe	Samara_Eneolithic	3	0	0	0	0.644	0	0	0	0	0	0.088	0.043	0.182	0	0	0	0	0.043	0	0	0	0
	Yamnaya_Kalmykia	6	0	0	0	0.551	0	0	0.064	0	0	0	0.026	0.266	0.054	0	0	0	0.039	0	0	0	0
	Yamnaya_Samara	9	0	0	0	0.498	0	0	0.049	0	0	0.014	0.042	0.268	0.062	0	0	0	0.066	0	0	0	0
	Afanasievo	5	0	0	0	0.526	0	0	0.023	0	0	0	0.053	0.272	0	0	0	0	0.126	0	0	0	0
	Poltavka	4	0	0	0	0.537	0	0	0.065	0	0	0	0.051	0.254	0	0	0	0	0.092	0	0	0	0
	Potapovka	3	0	0	0	0.546	0	0	0	0	0	0	0.063	0.256	0	0	0	0	0.135	0	0	0	0
Middle Neolithic to Copper Age Europe	Iberia_MN	4	0	0	0	0.043	0	0	0	0.093	0	0	0	0	0.130	0	0	0.021	0.668	0	0	0	0.045
	Central_MN	6	0	0	0	0.089	0	0	0	0.025	0	0	0	0	0.243	0	0	0	0.558	0	0	0	0.084
	Sweden	1	0	0	0	0.154	0	0	0	0.078	0	0	0	0	0.109	0	0	0	0.576	0	0	0	0.083
	Remedello	3	0	0	0	0.102	0	0	0	0.038	0	0	0	0	0.162	0	0	0	0.622	0	0	0	0.075
	Tyrol	1	0	0	0	0	0	0	0	0	0	0	0	0	0.292	0	0	0	0.579	0	0	0.044	0.086
	Iberia_Chalcolithic	12	0	0	0	0.075	0	0	0	0.069	0	0	0	0	0.055	0	0	0	0.801	0	0	0	0
	Spain	3	0	0.012	0	0.076	0	0	0	0.071	0	0	0	0	0.093	0	0.022	0	0.665	0	0	0	0.062
	Anatolia_ChL	1	0	0	0	0	0	0	0.096	0	0	0	0	0.115	0.324	0	0.047	0	0.268	0	0	0	0.150
Bronze Age Europe	Russia_EBA	1	0	0	0	0.680	0	0	0	0	0	0	0	0.320	0	0	0	0	0	0	0	0	0
	Northern_LNBA	10	0	0	0	0.495	0	0	0	0	0	0	0	0.094	0.057	0	0	0	0.354	0	0	0	0
	Central_LNBA	35	0	0	0	0.450	0	0	0.030	0	0	0	0	0.132	0.054	0	0	0	0.335	0	0	0	0
	Bell_Beaker_LN	17	0	0	0	0.460	0	0	0.017	0	0	0	0	0.047	0.040	0	0	0	0.436	0	0	0	0
	Hungary_BA	12	0	0	0	0.390	0	0	0	0	0	0	0	0	0.093	0	0	0	0.467	0	0.017	0	0.033
Late Bronze to Iron Age Steppe	Sintashta	5	0	0	0	0.553	0	0	0.058	0	0	0	0.021	0.149	0	0	0	0	0.219	0	0	0	0
	Andronovo	3	0	0	0	0.454	0	0	0.063	0	0	0	0.031	0.161	0	0	0	0.028	0.264	0	0	0	0
	Srubnaya	12	0	0	0	0.513	0	0	0.022	0	0	0	0.012	0.176	0.052	0	0	0	0.225	0	0	0	0
	Scythia	1	0.074	0	0	0.331	0	0	0.089	0	0.057	0	0	0.207	0	0	0	0	0.242	0	0	0	0
Western Asia	Georgia	2	0.027	0	0	0.049	0	0	0.090	0	0	0	0	0.377	0.458	0	0	0	0	0	0	0	0
	Iran_HotuIIIb	1	0	0	0	0	0	0	0.257	0	0	0	0	0.539	0	0	0	0.204	0	0	0	0	0
	Iran_N	4	0	0	0	0	0	0	0.043	0	0	0	0	0.741	0.216	0	0	0	0	0	0	0	0
	Iran_EN	4	0	0	0	0	0	0.018	0.059	0	0	0	0	0.533	0.264	0	0	0.095	0	0	0	0	0.031
	Iran_LN	1	0	0	0	0	0	0	0	0	0	0	0	0.539	0.308	0	0	0.153	0	0	0	0	0
	Iran_ChL	5	0	0	0	0	0	0	0.044	0.032	0	0	0	0.302	0.424	0	0	0.062	0	0	0	0	0.136
	Armenia_ChL	5	0	0	0	0.124	0	0	0.027	0	0	0	0	0.233	0.374	0	0	0.020	0.138	0	0	0	0.084
	Armenia_EBA	3	0	0	0	0	0	0	0.075	0	0	0	0	0.272	0.503	0	0	0	0.058	0	0	0	0.091
	Armenia_MLBA	1	0	0	0	0.181	0	0	0.087	0	0	0	0.042	0.300	0.391	0	0	0	0	0	0	0	0
	Iran_IA	1	0	0	0	0.084	0	0	0	0	0	0	0	0.310	0.427	0	0	0	0	0	0	0	0.179
	Iran_recent	1	0	0	0	0.113	0	0	0	0	0	0	0	0.285	0.392	0	0	0	0	0	0.054	0	0.155
	Natufian	6	0	0	0.068	0	0	0	0	0.212	0	0	0	0	0.108	0	0	0	0	0	0	0	0.612
	Levant_N	13	0	0	0	0	0	0	0	0.161	0	0	0	0	0.121	0	0	0	0	0	0	0	0.718
	Levant_BA	3	0	0	0	0	0	0	0	0.079	0	0	0	0.075	0.323	0	0	0	0.081	0	0.018	0	0.424

**Table 2:** Y chromosome and mitochondrial DNA haplogroups for 49 samples of ancient Eurasians.

**Group**	**Sample**	**Y Haplogroups**	**Mitochondrial Haplogroups**
European hunter-gatherers	Western	1 C1a, 2 I2a	1 R1, 2 U5
	Switzerland	1 I2	1 U5
	Sweden	1 I2a	1 U4
	Motala	3 I2a, 1 I2c	2 U2, 4 U5
	Eastern	1 J, 1 R1a, 1 R1b	1 C1, 1 U4, 1 U5
Early Neolithic	Anatolia	1 G2a	1 K1, 1 X2
	Anatolia_Neolithic	1 C1a, 1 G, 7 G2a, 1H, 2 H2, 1 I, 1 I2c, 1 J2a	1 H, 1 H5, 2 J1, 9 K1, 3 N1, 2 T2, 2 U3, 1 U8, 1 W1, 2 X2
	Macedonia_EN	NA	1 X2
	Macedonia_LN	1 G2a	1 J1, 1 K1
	Hungary_EN	2 C1a, 1 H2, 1 I2a	1 H, 2 J1, 2 K1, 3 N1, 1 U5, 1 X2
	LBK_EN	4 G2a, 2 T1a	1 H1, 1 H46, 1 J1, 2 K1, 3 N1, 6 T2, 1 X2
	Iberia_EN	1 I2a, 1 R1b	1 J1, 1 N1, 1 T2, 1 V
Eneolithic to Middle Bronze Age steppe	Samara_Eneolithic	1 Q1a, 1 R1a, 1 R1b	1 H2, 1 U4, 1 U5
	Yamnaya_Kalmykia	1 I2a, 4 R1b	2 T2, 1 U4, 3 U5
	Yamnaya_Samara	7 R1b	1 H2, 1 H6, 1 H13, 1 T2, 1 U4, 2 U5, 1 W3, 1 W6
	Afanasievo	NA	2 J2, 1 T2, 2 U5
	Poltavka	4 R1b	1 H6, 1 H13, 1 I3, 1 U2
	Potapovka	1 P1, 1 R1a	1 C1, 1 T1, 1 U2
Middle Neolithic to Copper Age Europe	Iberia_MN	1 H2, 1 I2a	1 H1, 2 K1, 1 U5
	Central_MN	1 G2a, 1 I, 1 I2a, 1 R	1 H, 1 H1, 1 HV, 2 T2, 1 U3
	Sweden	NA	1 H1
	Remedello	3 I	1 H2, 1 J1, 1 X2
	Tyrol	1 G2a	1 K1
	Iberia_Chalcolithic	1 G2a, 2 I, 3 I2a	1 H1, 4 H3, 1 J1, 1 J2, 3 K1, 1 U3, 1 X2
	Spain	1 H2, 1 I2a	1 H3, 1 U5, 1 X2
	Anatolia_ChL	NA	1 K1
Bronze Age Europe	Russia_EBA	1 R1b	1 N1
	Northern_LNBA	3 I, 2 R1a, 3 R1b	1 H3, 1 I, 1 J1, 3 K1, 1 K2, 1 T1, 2 T2
	Central_LNBA	1 CT, 1 I2, 1 I2c, 1 K(xLT), 2 P1, 8 R1a, 1 R1b	1 H1, 1 H3, 1 H4, 1 H5, 1 H23, 1 HV, 1 I3, 5 J1, 1 J2, 3 K1, 1 K2, 1 T1, 3 T2, 3 U4, 7 U5, 1 V9, 1 W3, 1 W6, 1 X2
	Bell_Beaker_LN	1 R1, 5 R1b	2 H, 3 H1, 1 H2, 1 H3, 1 H5, 1 H13, 1 H44, 1 H46, 1 J1, 2 K1, 2 U5, 1 W1
	Hungary_BA	1 G2a, 1 I, 2 I2a, 1 J2a	1 H2, 1 H11, 1 J1, 3 K1, 1 T1, 3 T2, 2 U5
Late Bronze to Iron Age steppe	Sintashta	2 R1a	1 J1, 1 J2, 1 N1, 2 U2
	Andronovo	NA	1 U2, 2 U4
	Srubnaya	6 R1a	1 H2, 1 H3, 1 H5, 1 H6, 1 I1, 1 J2, 1 K1, 1 T1, 1 T2, 3 U5
	Scythia	1 R1a	1 G2
Western Asia	Georgia	1 J, 1 J2	1 H13, 1 K3
	Iran_HotuIIIb	1 J	1 HV2
	Iran_N	1 CT, 1 P1	1 J1, 1 R2, 1 X2
	Iran_EN	1 G2b	1 J1, 2 R2, 1 T2
	Iran_LN	1 G2a	1 K1
	Iran_ChL	1 G1a, 1 J	1 H29, 1 I1, 1 K1, 1 U3, 1 U7
	Armenia_ChL	3 L1a	1 H, 1 H2, 2 K1, 1 U4
	Armenia_EBA	1 R1b	1 H1, 1 U3, 1 X2
	Armenia_MLBA	NA	1 T1
	Iran_IA	1 R1b	1 N1
	Iran_recent	NA	1 U1
	Natufian	2 CT, 1 E1b1, 2 E1b1b1b2	2 J2, 1 N1
	Levant_N	3 CT, 1 E, 2 E1b1b1, 1 H2, 1 T	1 I, 3 K1, 2 R0, 4 T1
	Levant_BA	1 J, 1 J1	1 H14, 1 R0, 1 X2

### Early neolithic peoples

Second, I considered samples from Anatolia (Anatolia and Anatolia_Neolithic) and Macedonia (Macedonia_EN and Macedonia_LN), as well as Early Neolithic samples from Hungary (Hungary_EN), Germany (LBK_EN), and Iberia (Iberia_EN), collectively referred to early European farmers (Figure 1B). These 58 individuals averaged 47.2% Southern European, 31.9% Western Asian, 14.2% Arabian, and 6.8% Northern African ancestries (Table 1). Y DNA haplogroups included 14 G, four H, four I, three C, two T, one J, and one R1b (Table 2). Multiple descendants of the mitochondrial haplogroup R (H, V, J, T, U, and K) accounted for 70.7% of lineages (Table 2).

### Eneolithic to middle Bronze age steppe peoples

Third, I considered the Eneolithic to Middle Bronze Age steppe peoples (Figure 1C). The Samara_Eneolithic sample had 64.4% Northern European, 18.2% Southern Asian, 8.8% Circumpolar, 4.3% Amerindian, and 4.3% Southern European ancestries (Table 1). The 27 Early to Middle Bronze Age steppe individuals (Yamnaya_Kalmykia, Yamnaya_Samara, Afanasievo, Poltavka, and Potapovka) averaged 54.7% Northern European, 27.8% Southern Asian, 7.9% Southern European, 4.7% Kalash, 4.2% Amerindian, and 0.8% Western Asian ancestries (Table 1). I included the Potapovka sample here because the sum of absolute differences in ancestry was greater post-Potapovka rather than post-Poltavka. In these steppe peoples, 76.2% of Y DNA haplogroups were R1b and 86.7% of mitochondrial haplogroups were H, J, T, or U (Table 2).

### Middle neolithic to copper age European peoples

Fourth, I considered the Middle Neolithic to Copper Age European peoples (Figure 1D). The 10 Middle Neolithic individuals (Iberia_MN and Central_MN) averaged 64.2% Southern European, 18.2% Western Asian, 6.2% Northern European, 6.2% Arabian, 4.3% Northern African, and 0.9% Oceanian ancestries (Table 1). Five of six Y DNA haplogroups were G, H, or I; the one other haplogroup was R (Table 2). Mitochondrial haplogroups included H, HV, K, T, and U (Table 2). The 21 Copper Age individuals (Sweden, Remedello, Tyrol, Iberia_Chalcolithic, Spain, and Anatolia_ChL) averaged 71.8% Southern European, 10.9% Western Asian, 7.6% Northern European, 5.6% Northern African, and 4.2% Arabian ancestries (Table 1). Y DNA haplogroups were 75.0% I (Table 2). This time period includes the Tyrolean Iceman who had 57.9% Southern European and 29.2% Western Asian ancestries (Table 1), the latter of which is consistent with his Y DNA haplogroup G2a (Keller et al., 2012). Mitochondrial haplogroups were 85.7% H, U, K, or J (Table 2). The transition from Middle Neolithic to Copper Age involved the acquisition of 7.6% Southern European, 1.4% Northern European, and 1.3% Northern African ancestries.

### Bronze age European peoples

The 75 Bronze Age individuals (Russia_EBA, Northern_LNBA, Central_LNBA, Bell_Beaker_LN, and Hungary_BA) averaged 49.0% Northern European, 40.0% Southern European, 5.9% Western Asian, and 5.1% Southern Asian ancestries (Figure 1E and Table 1). The transition from the Copper Age to the Bronze Age involved the acquisition of 41.4% Northern European and 5.1% Southern Asian ancestries. Y DNA haplogroups were 60.0% R (10 R1a and 10 R1b), while the mitochondrial haplogroups remained 90.7% H, V, K, U, J, or T (Table 2).

### Late Bronze to iron age steppe peoples

The 21 Late Bronze to Iron Age steppe individuals (Sintashta, Andronovo, Srubnaya, and Scythia) had 50.9% Northern European, 23.9% Southern European, 17.7% Southern Asian, 3.4% Kalash, 2.5% Western Asian, and 1.6% Amerindian ancestries (Figure 1F and Table 1). Thus, post-Potapovka, population change in the steppes involved an increase of 16.0% Southern European and 1.7% Western Asian ancestries (a ratio of 9.4) and a decrease of 10.1% Southern Asian, 3.8% Northern European, and 2.6% Amerindian ancestries. This change does not fit with gene flow of people like the Early Neolithic peoples (Mathieson et al., 2015), who had a ratio of Southern European to Western Asian ancestry of 1.5; the source is more consistent with European Copper or Bronze Age peoples, in whom the ratios were 6.6 and 6.8, respectively. All nine Y DNA haplogroups were R1a, while 85.7% of mitochondrial haplogroups were H, V, U, K, J, or T (Table 2).

### Western Asian peoples

The two Georgian hunter-gatherers did not group with the European hunter-gatherers (Figure 1G). The Georgian hunter-gatherers averaged 45.8% Western Asian and 37.7% Southern Asian ancestries, with only 4.9% Northern European and no Southern European ancestries (Table 1). Both Y DNA haplogroups were J (Table 2). The mitochondrial haplogroups were H and K; neither matriline was observed in the European hunter-gatherers (Table 2).

The ancient Iranians were characterized through the Late Neolithic period by predominantly Southern Asian ancestry (Figure 1G and Table 1). The proportion of Western Asian ancestry doubled through the Iron Age. The ancient Armenians resembled the Georgian hunter-gatherers in having a mixture of Western Asian and Southern Asian ancestries (Figure 1G and Table 1). In the ancient Armenians relative to the Georgian hunter-gatherers, Northern European, Southern European, and Arabian ancestries increased in the Copper Age.

The Natufian sample consisted of 61.2% Arabian, 21.2% Northern African, 10.9% Western Asian, and 6.8% Omotic ancestry (Figure 1G and Table 1). The transition in the Levant from the Epipaleolithic to the Neolithic period involved an increase of Arabian ancestry at the expense of Northern African and Omotic ancestries. The transition from the Neolithic period to the Bronze Age involved the acquisition of principally Western Asian ancestry, with smaller contributions of Southern European and Southern Asian ancestries.

## Discussion

Using a large, global reference panel, I found more population structure than previously reported among 279 ancient Eurasians. All samples showed multiple autosomal ancestries, Y DNA haplogroups, and mitochondrial haplogroups. Given such a large amount of ancestral heterogeneity, previous estimates of allele frequencies, including claims of natural selection, may have been confounded by this unrecognized population structure.

In contrast to previous reports, Western and Eastern hunter-gatherers were not homogeneous for different ancestries (Lazaridis et al., 2016) nor were they separated (Gallego-Llorente et al., 2016). Amerindian, Circumpolar, and Southern Asian ancestries existed in Eastern and, to a lesser extent, Scandinavian hunter-gatherers thousands of years before the European Bronze Age and in higher proportions than in Bronze Age steppe populations. Amerindian and Circumpolar ancestries were absent from Europeans from the Early Neolithic through the Bronze Age. These results are consistent with a shared relationship predating the Neolithic.

The transition from Eneolithic to Early and Middle Bronze Age steppe peoples involved increases in Southern Asian and Southern European ancestries that do not fit with a European hunter-gatherer source (Mathieson et al., 2015) and more broadly do not fit with any of the samples, suggesting an unknown source population. Currently, Southern Asian ancestry co-localizes with Y DNA haplogroup L and correlates with Indo-Iranian languages (Baker et al., 2017). Although there are no L haplogroups in any of these Early to Middle Bronze Age steppe individuals, the correlation with Indo-Iranian languages strengthens the connection between Early to Middle Bronze Age steppe peoples and the introduction of Indo-European languages into Europe.

Northern and Southern European ancestries were primarily associated with Y haplogroup I from before the Neolithic until the Copper Age. A low level of Y DNA haplogroup R was present in Europe prior to and during the Neolithic. In the Bronze Age, an increase in the proportion of Y haplogroup R while the distribution of mitochondrial haplogroups remained essentially unchanged is consistent with male-biased gene flow (Goldberg et al., 2017). Collectively, the findings suggest two male-biased migrations from the steppes to Europe, rather than one prolonged event (Goldberg et al., 2017). The first event was associated with Eneolithic to Middle Bronze Age steppe peoples located north and east of the Black Sea and characterized by R1b; this incoming ancestry is associated with present-day Southern European ancestry. The second event was associated with Late Bronze Age steppe peoples located north and east of the Caspian Sea and characterized by R1a; this incoming ancestry is associated with present-day Northern European ancestry. There was also gene flow from Europe to the steppes associated with the transition from the Middle to Late Bronze Age.

In the Copper Age, Northern African ancestry increased while Arabian ancestry decreased, possibly indicating entry into Europe from northwest Africa rather than northeast Africa. The ratio of 5.4-fold more Southern European than Northern European ancestries and the presence of Northern African ancestry acquired from the Early Neolithic to the Copper Age are inconsistent with a resurgence of peoples related to Western hunter-gatherers, given that Western hunter-gatherers had 1.6-fold more Northern European than Southern European ancestry and no Northern African ancestry. Instead, this ancestral profile is suggestive of an expansion of peoples from Southern Europe resembling those from the Remedello culture.

In the ancient Iranians, the proportion of Western Asian ancestry doubled through the Iron Age, suggesting gene flow from the Caucasus rather than the Levant (Lazaridis et al., 2016), while smaller amounts of Arabian and South Indian ancestries suggest gene flow from the west and the east, respectively. In the ancient Armenians, Northern European, Southern European, and Arabian ancestries increased in the Copper Age, again suggesting gene flow from multiple directions. In the Levant, Lazaridis et al. (2016) suggested that the transition from the Neolithic period to the Bronze Age resulted from admixture from people resembling Chalcolithic Iranians. This putative source is unlikely because none of the ancient Iranian samples had Southern European ancestry; a Caucasian source, such as the Chalcolithic or Early Bronze Age Armenians, provides a better fit.

The Early Neolithic samples, i.e., early farmers, qualitatively differed from hunter-gatherers by harboring more diverse sets of Y DNA haplogroups and mitochondrial lineages. This result suggests that the initial spread of agriculture occurred by demic diffusion involving both males and females. The early European farmers had no Southern Asian ancestry, which does not support an origin in the eastern part of Western Asia, i.e., present-day Iran. However, ancient Western Asian peoples and early European farmers shared Western Asian ancestry, and thus were not genetically dissimilar (Gallego-Llorente et al., 2016). The increase of Western Asian ancestry in the Bronze Age Levant and throughout Neolithic Western Asia is consistent with demic diffusion of agriculture via a single origin, with the original people characterized by Western Asian ancestry. Even if farming was introduced into Europe by such individuals, then subsequent migrations of semi-nomadic pastoralists from the steppes suggests that the ultimate spread of agriculture occurred by cultural, not demic, diffusion.

Previously, no significant sharing of ancestral components with sub-Saharan African populations was found to accompany the presence of Y haplogroup E1b1b1b2 (Lazaridis et al., 2016). E1b1b1b1a-M81, not E1b1b1b2-Z830, is presently common among Berbers in North Africa (Arredi et al., 2004; Trombetta et al., 2015). E1b1b1b1a-M81 has a time to most recent common ancestor of only 2,300 (95% confidence interval [1900, 2700]) years before present (Urasin, 2017) and therefore was not prevalent in Northern African ancestry during the Epipaleolithic. Ancestry shared by Omotic-speaking peoples is found predominantly in present-day southern Ethiopia and is associated with haplogroup E, thus revealing a plausible source.

Using TreeMix (Pickrell and Pritchard, 2012) to reconstruct migration graphs from ancestries inferred by ADMIXTURE, we previously observed that Southern European and Northern European ancestries clustered with 77% probability and that Southern European and Arabian ancestries clustered with 23% probability (Shriner et al., 2014). We hypothesized that the primary mode reflected the relationship between R1a, characteristic of present-day Northern European ancestry, and R1b, characteristic of present-day Southern European ancestry. We further hypothesized that the secondary mode reflected the relationship between I2, present in lower frequencies in present-day Southern European ancestry, and J (more precisely, J1), characteristic of Arabian ancestry. The current findings support both hypotheses. The fact that Southern European ancestry experienced a replacement of haplogroup I by haplogroup R and yet was inferred by ADMIXTURE to be one ancestry, rather than two distinct ancestries, serves as a strong caveat in the interpretation of ancestries, while TreeMix could detect both stages of Southern European ancestry.

All ancestries in our reference panel were estimated from present-day individuals and therefore reflect present-day ancestry-specific allele frequencies. As these allele frequencies change through evolutionary time, it is possible to relate ancestries phylogenetically and make inferences about the common ancestors of ancestries. Projecting ancient individuals onto present-day ancestries will lead to increasingly incorrect inference as the age of the ancient individual increases. Thus, this issue is a bigger problem for Ice Age Europeans than for Bronze Age Europeans. This problem can be solved if allele frequencies for each of the ancestors of the present-day ancestries were known.

In summary, rather than three (Lazaridis et al., 2014) or four (Jones et al., 2015; Lazaridis et al., 2016) ancestral populations, I found considerably more population structure across 279 ancient Eurasians, involving a total of 18 autosomal ancestries, 13 Y DNA haplogroups, and 14 mitochondrial haplogroups, such that no sample was ancestrally homogeneous. Even if ancestries are inferred from extant individuals, ancestry analysis can provide historical insight in the absence of ancient DNA samples. Perhaps most importantly, using a consistent, unified nomenclature will enhance research of both ancient and present-day peoples.

## Author contributions

DS designed the study, performed the research, interpreted the results, and wrote the manuscript.

### Conflict of interest statement

The author declares that the research was conducted in the absence of any commercial or financial relationships that could be construed as a potential conflict of interest.
