# Supplementary material for: Re-analysis of Whole Genome Sequence Data From 279 Ancient Eurasians Reveals Substantial Ancestral Heterogeneity
Source: Front Genet. 2018 Jul 20;9:268. doi: 10.3389/fgene.2018.00268 (PMC6062619; doi:10.3389/fgene.2018.00268)
Supplement: Supplementary file 2 [file Data_Sheet_1.DOCX]

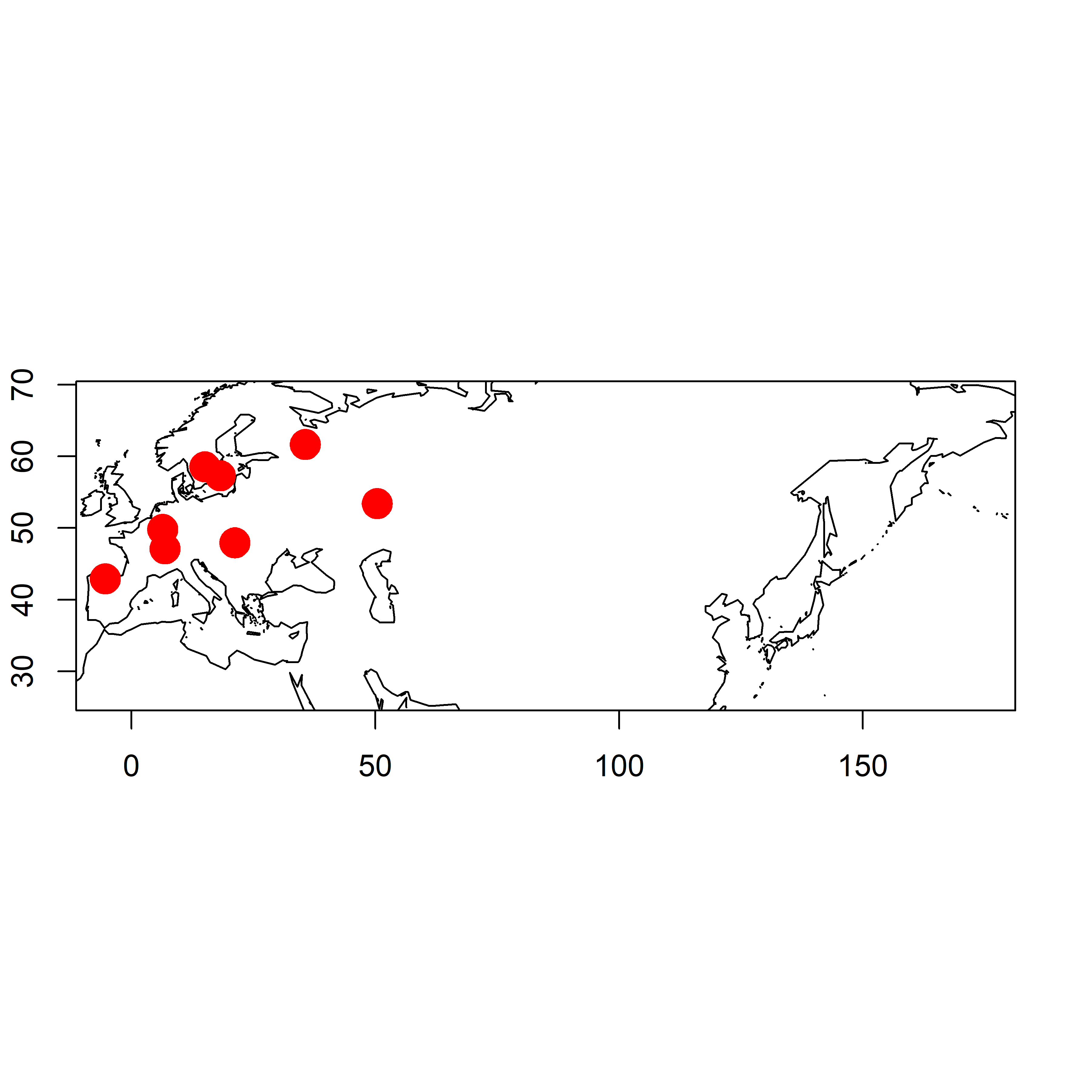


Figure S1. (A) Sampling locations of European hunter-gatherers.


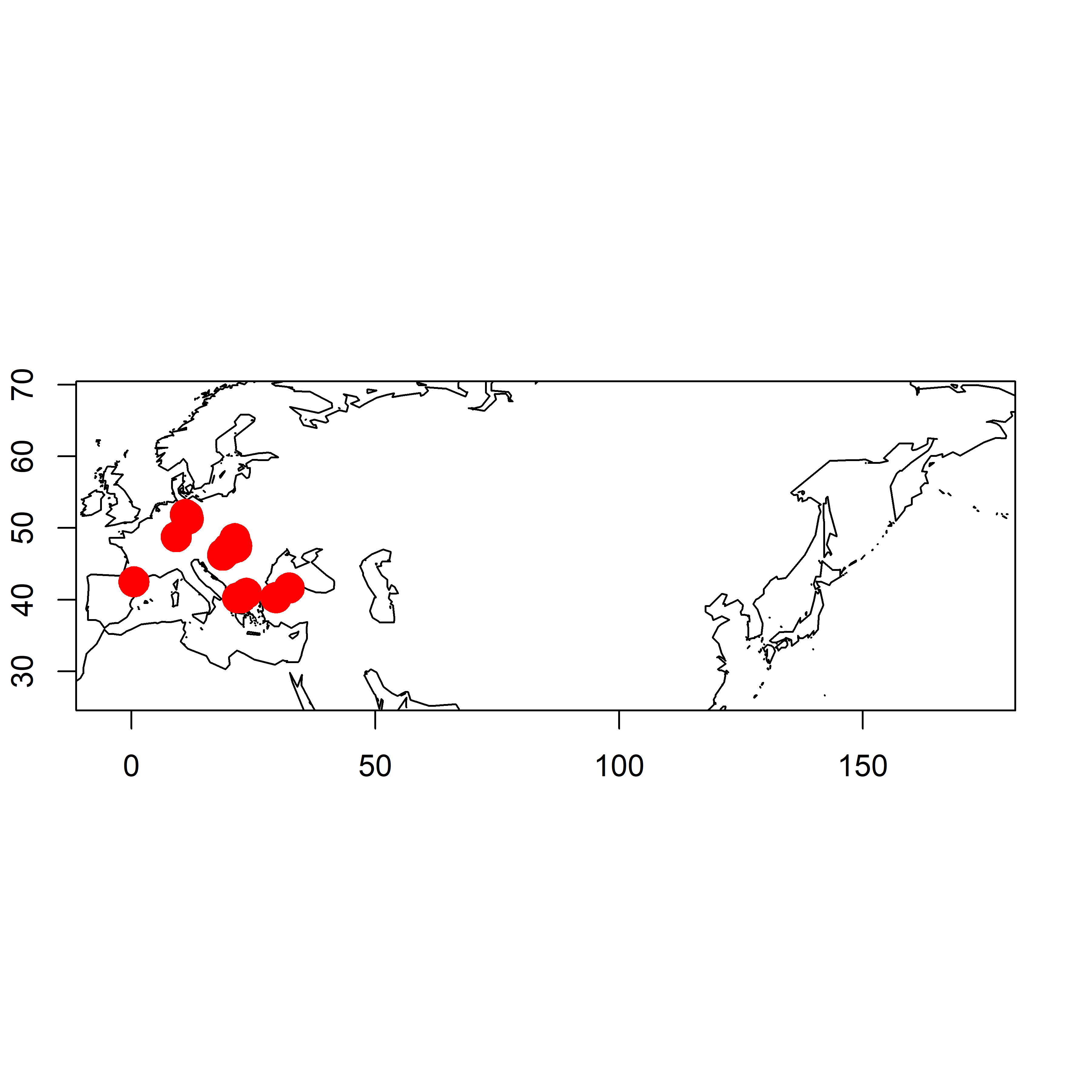


Figure S1. (B) Sampling locations of early Neolithic peoples.


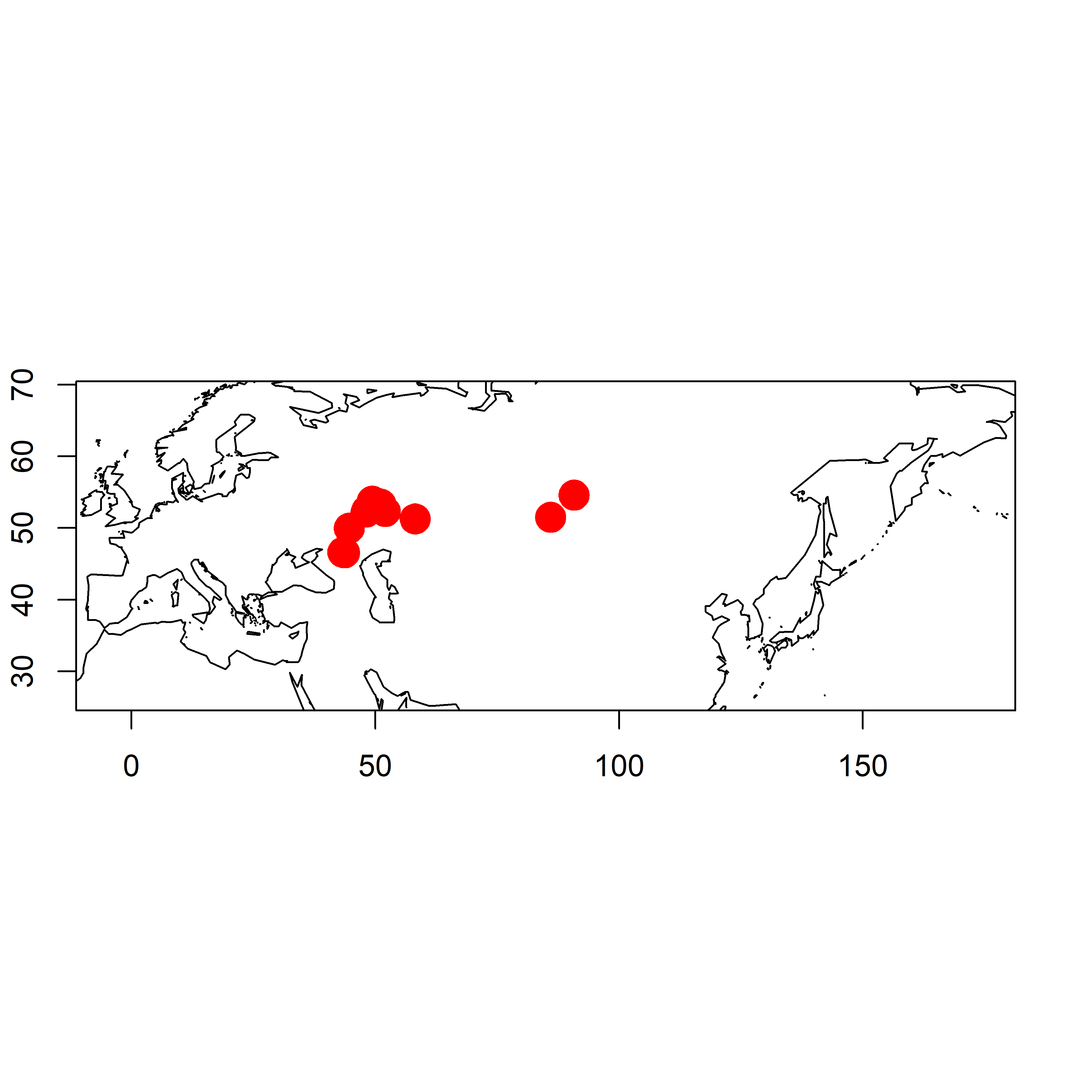


Figure S1. (C) Sampling locations of Eneolithic to Middle Bronze Age steppe peoples.


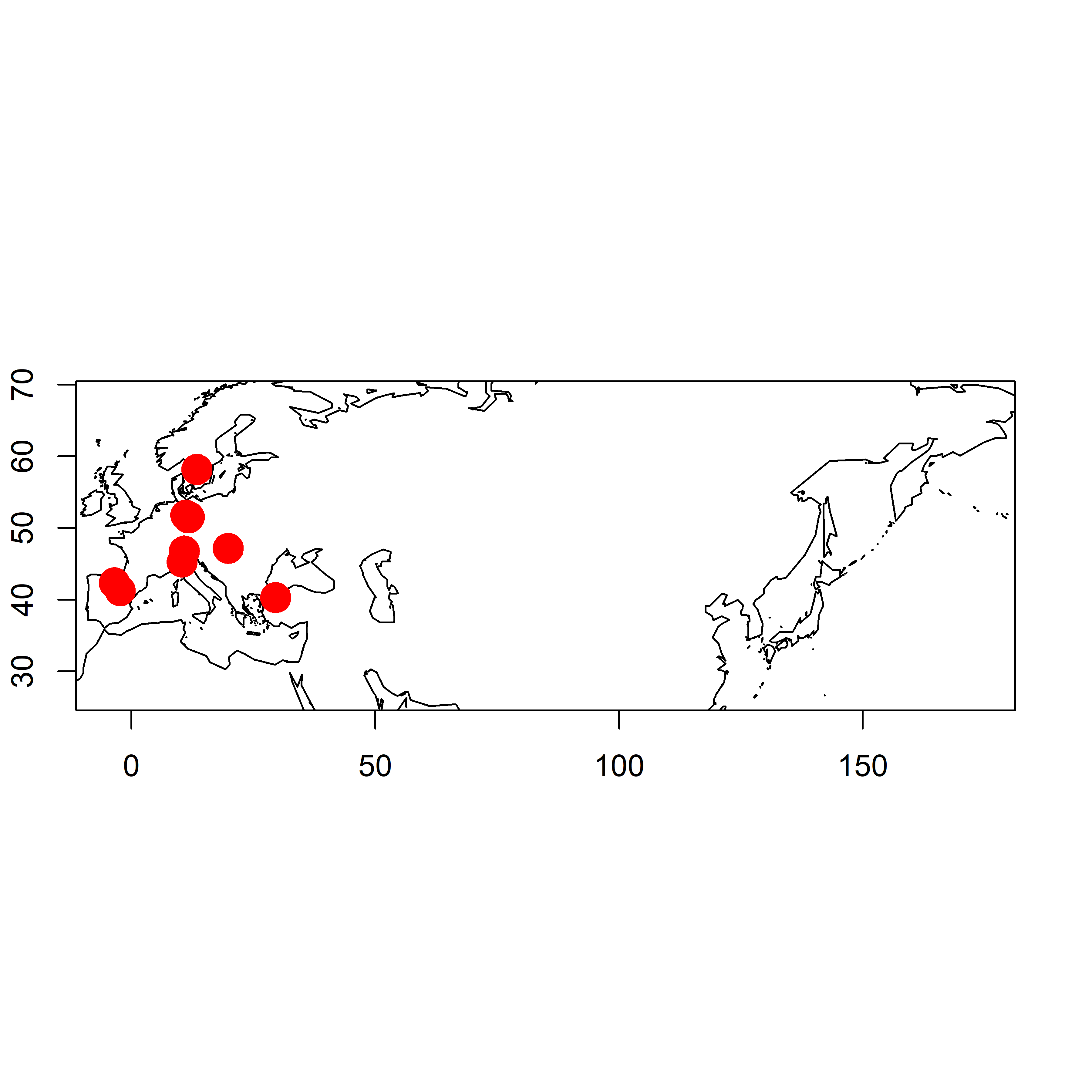


Figure S1. (D) Sampling locations of Middle Neolithic to Copper Age European peoples.


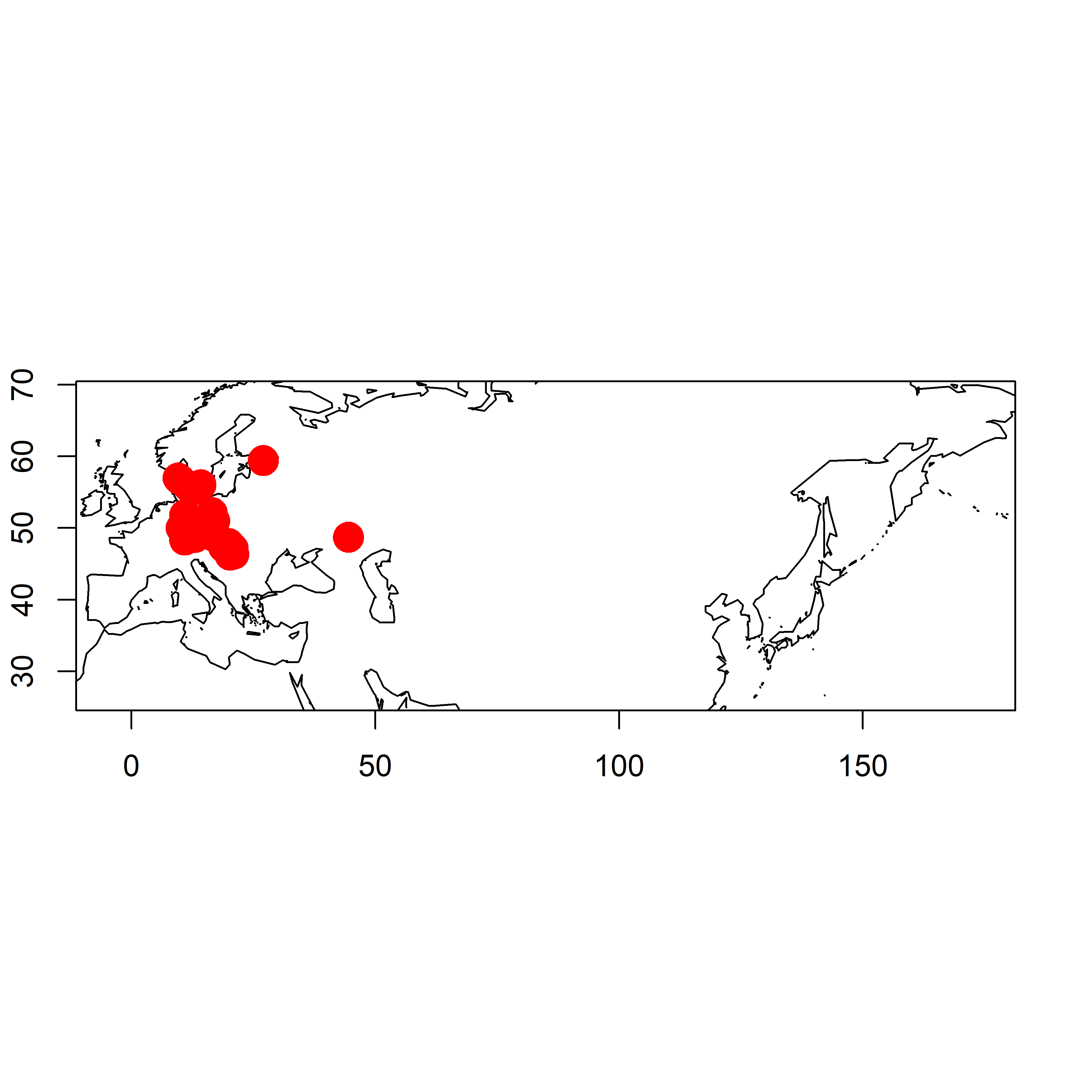


Figure S1. (E) Sampling locations of Bronze Age European peoples.


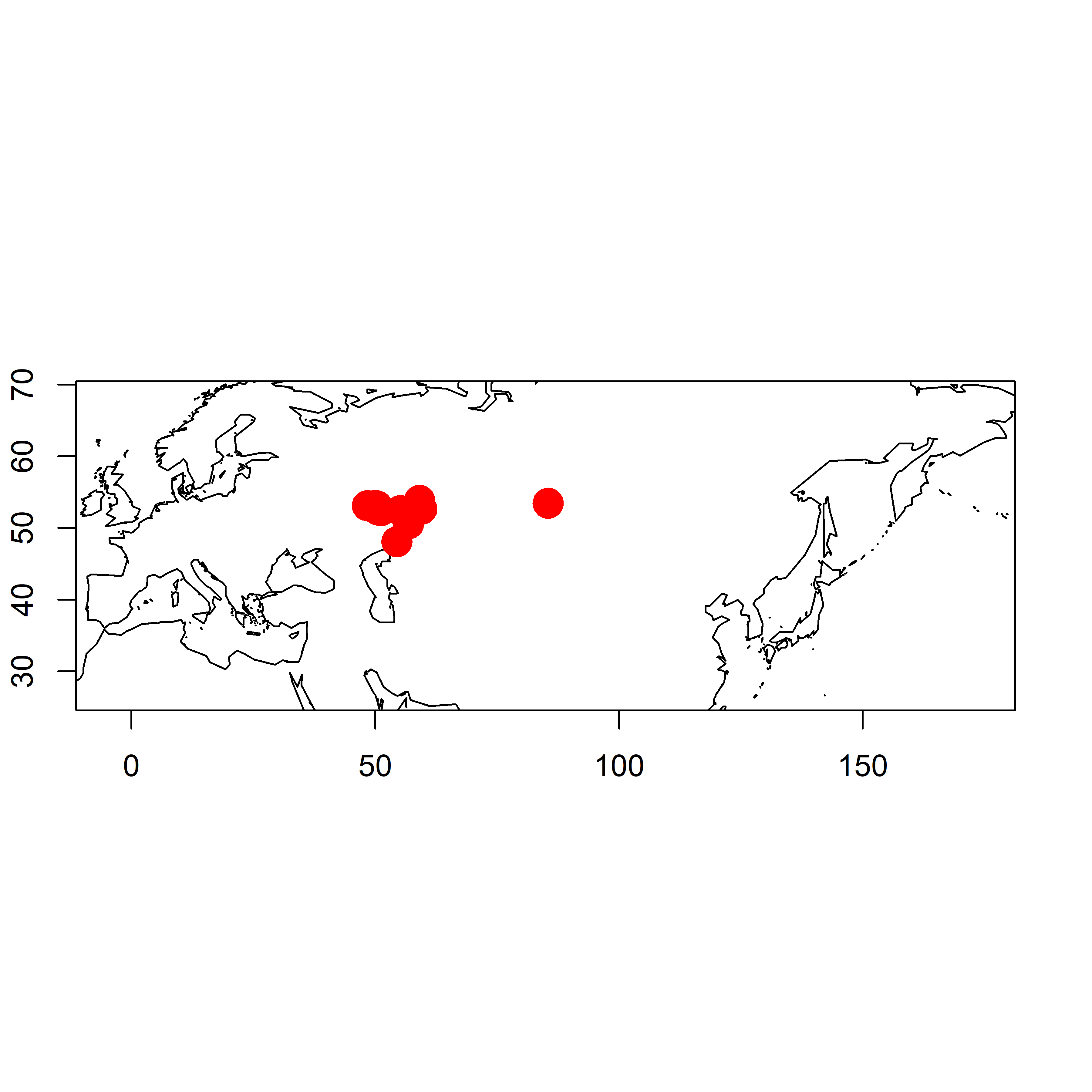


Figure S1. (F) Sampling locations of Late Bronze to Iron Age steppe peoples.


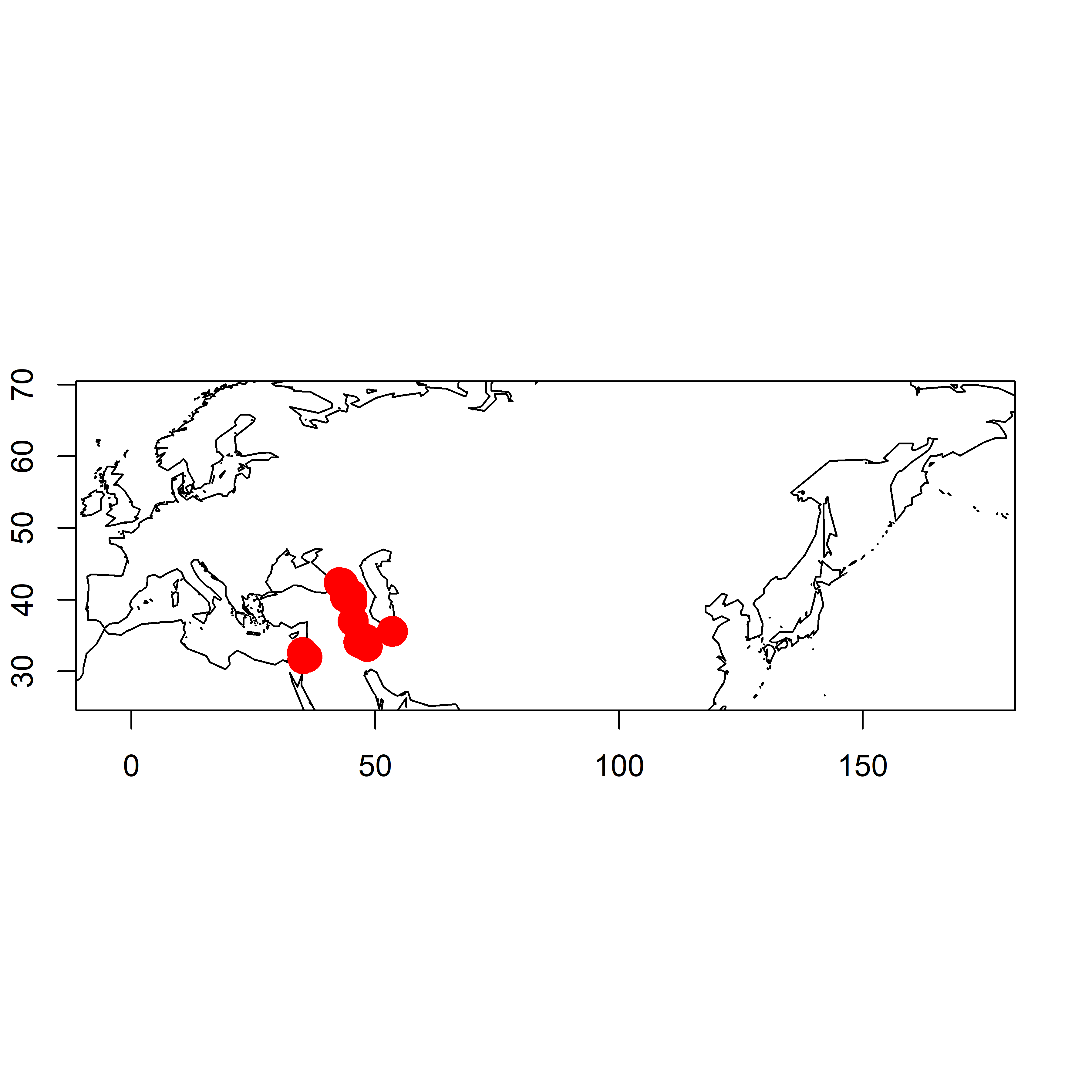


Figure S1. (G) Sampling locations of Western Asian peoples.
