# Supplementary material for: Re-analysis of Whole Genome Sequence Data From 279 Ancient Eurasians Reveals Substantial Ancestral Heterogeneity
Source: Front Genet. 2018 Jul 20;9:268. doi: 10.3389/fgene.2018.00268 (PMC6062619; doi:10.3389/fgene.2018.00268)
Supplement: Supplementary file 3 [file Data_Sheet_2.DOCX]

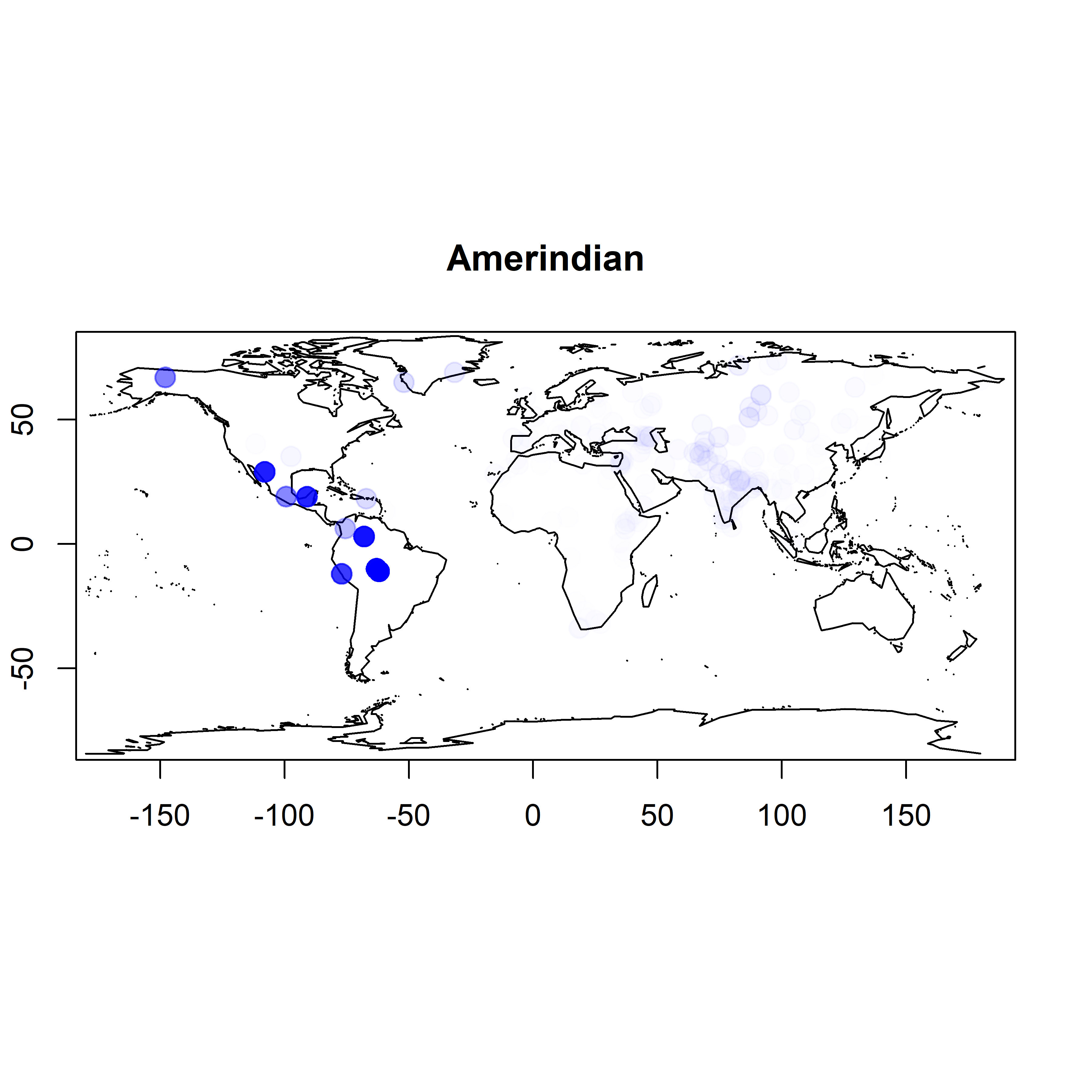


Figure S2. (A) Present-day geographic distribution of Amerindian ancestry. The intensity of blue is directly proportional to the denoised and renormalized sample mean.


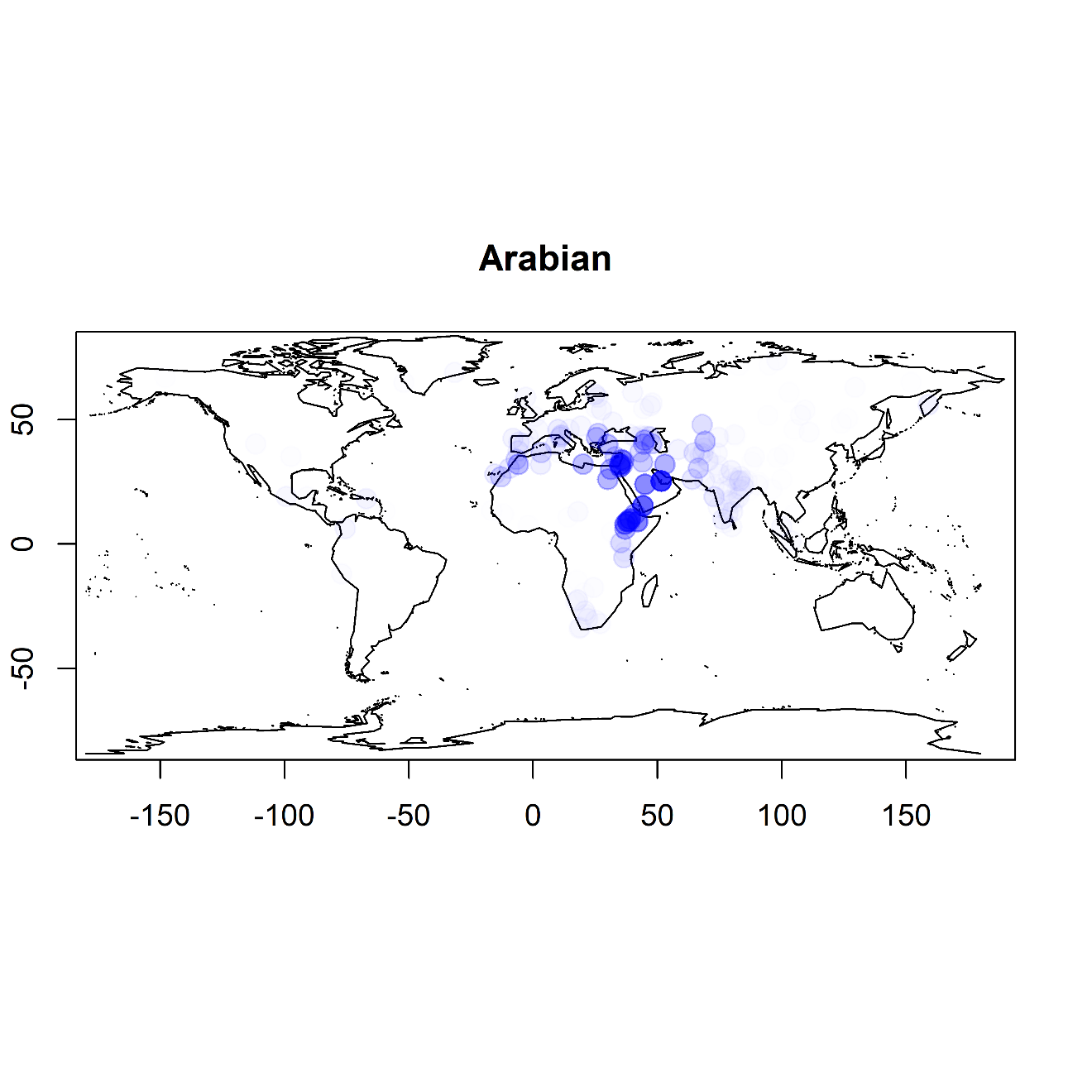


Figure S2. (B) Present-day geographic distribution of Arabian ancestry. The intensity of blue is directly proportional to the denoised and renormalized sample mean.


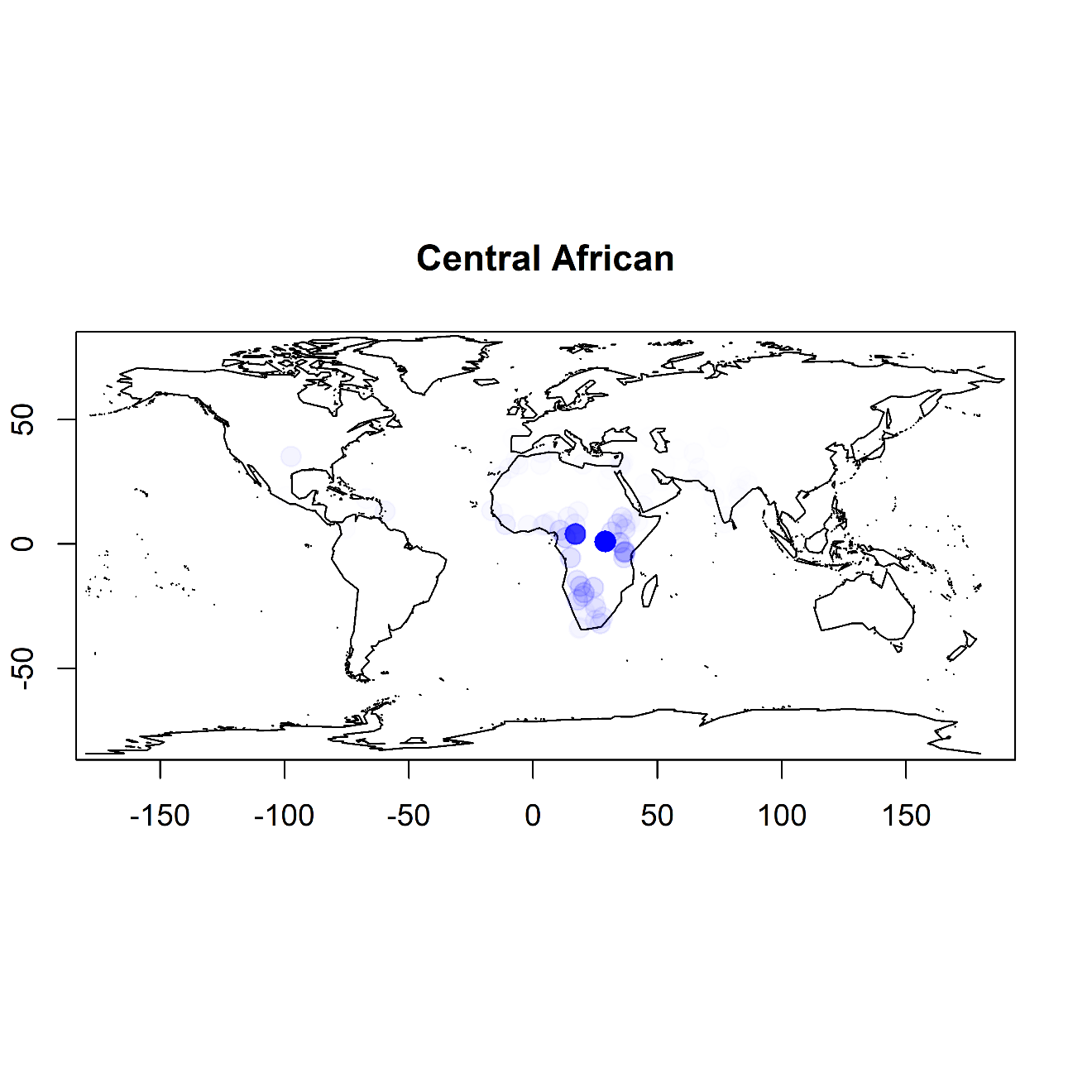


Figure S2. (C) Present-day geographic distribution of Central African ancestry. The intensity of blue is directly proportional to the denoised and renormalized sample mean.


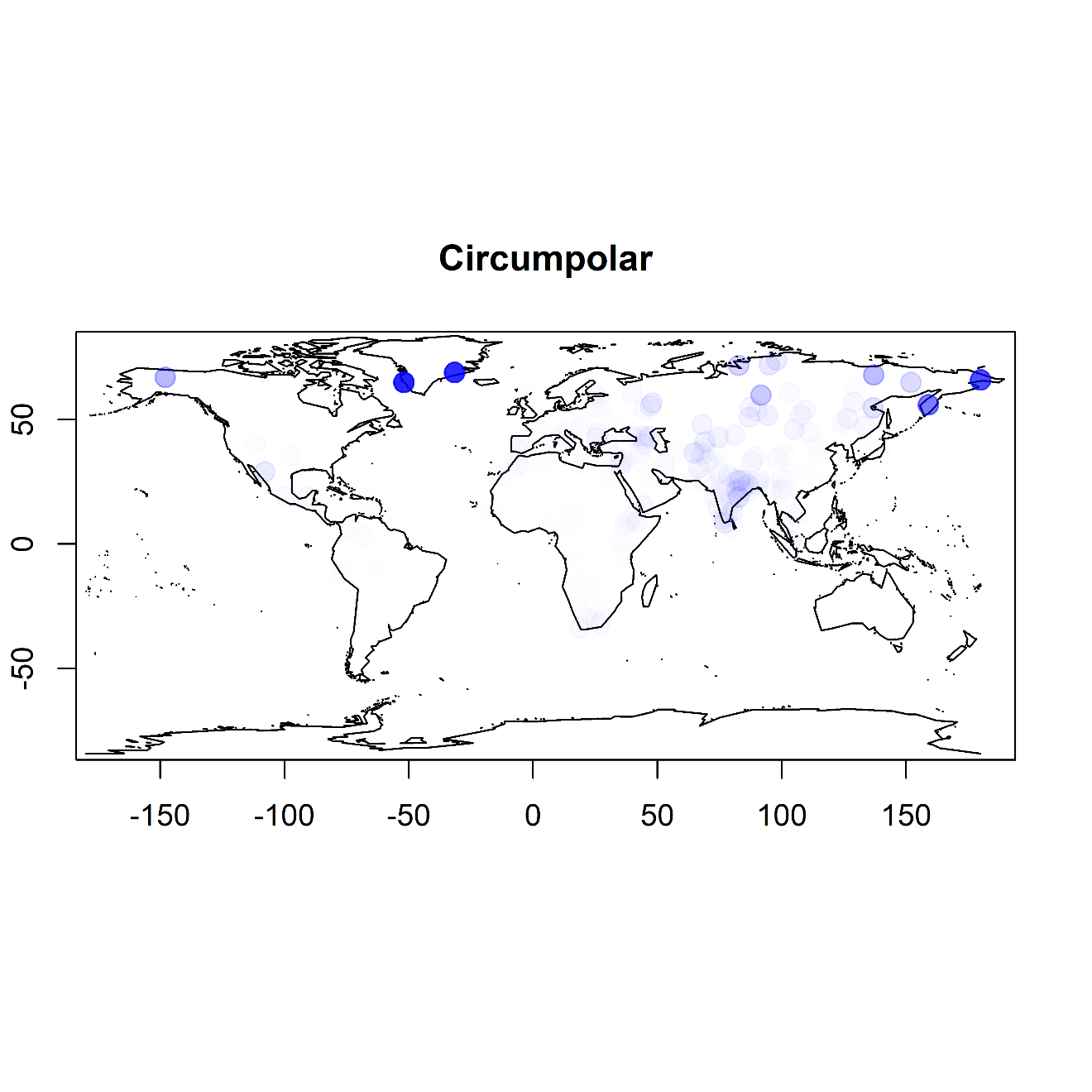


Figure S2. (D) Present-day geographic distribution of Circumpolar ancestry. The intensity of blue is directly proportional to the denoised and renormalized sample mean.


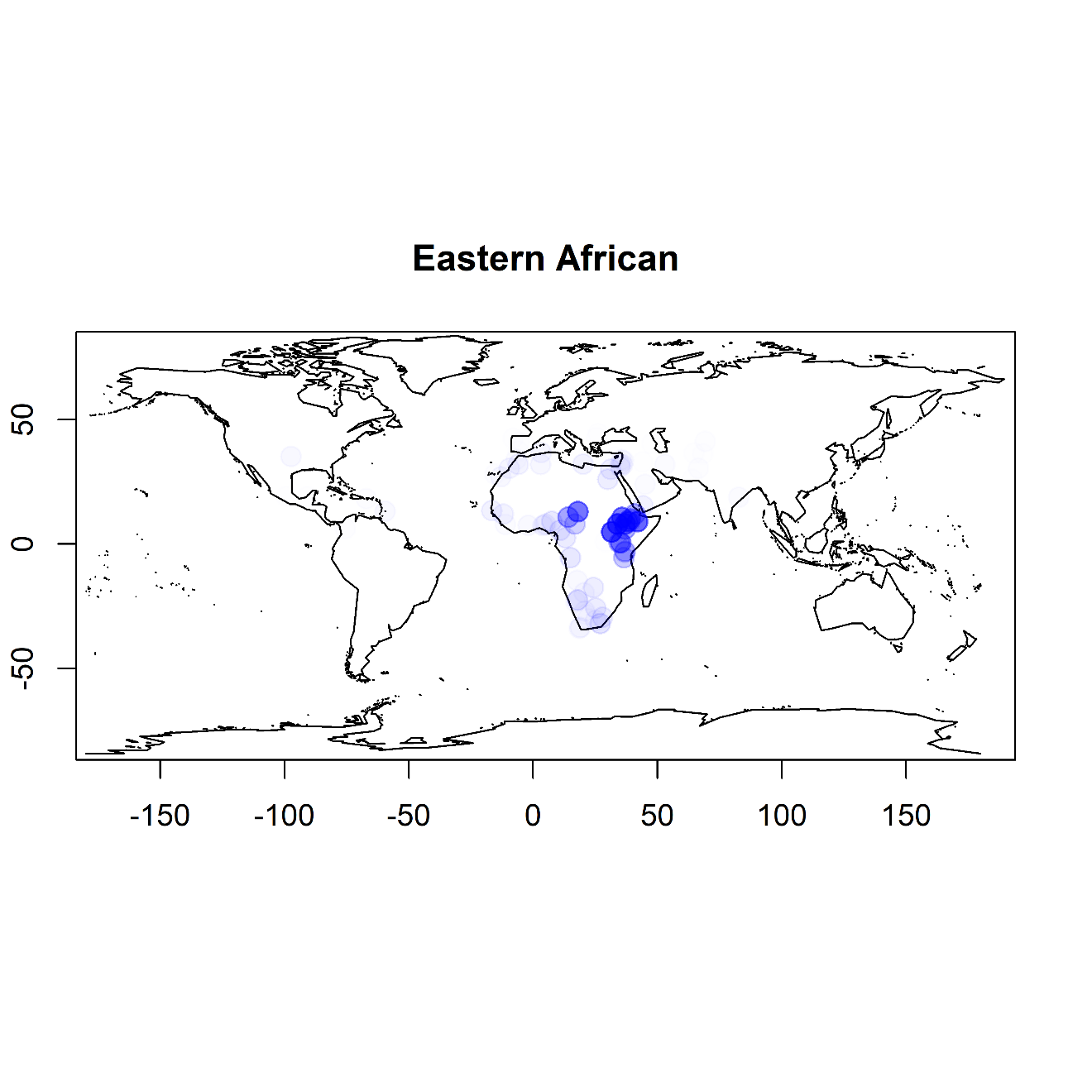


Figure S2. (E) Present-day geographic distribution of Eastern African ancestry. The intensity of blue is directly proportional to the denoised and renormalized sample mean.


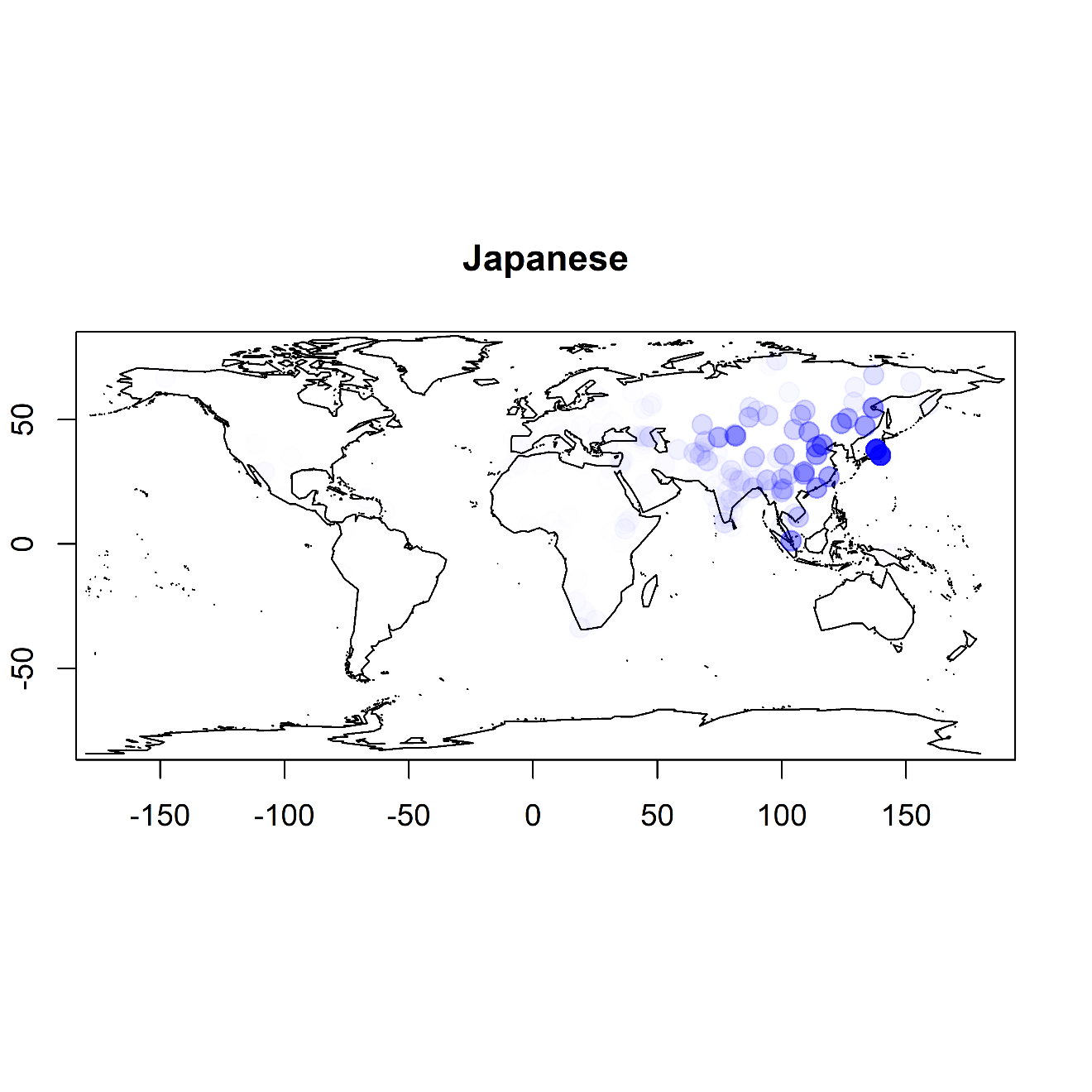


Figure S2. (F) Present-day geographic distribution of Japanese ancestry. The intensity of blue is directly proportional to the denoised and renormalized sample mean.


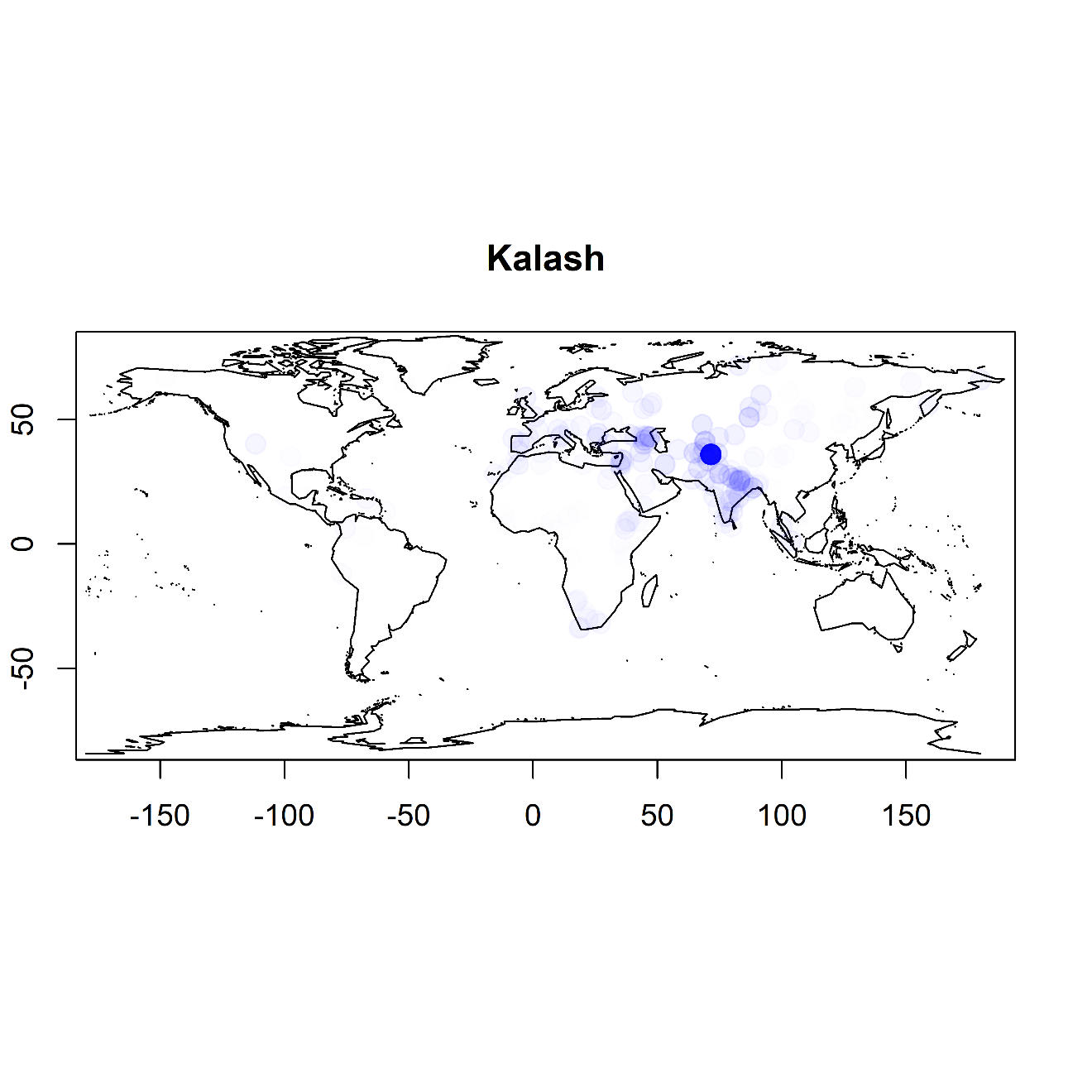


Figure S2. (G) Present-day geographic distribution of Kalash ancestry. The intensity of blue is directly proportional to the denoised and renormalized sample mean.


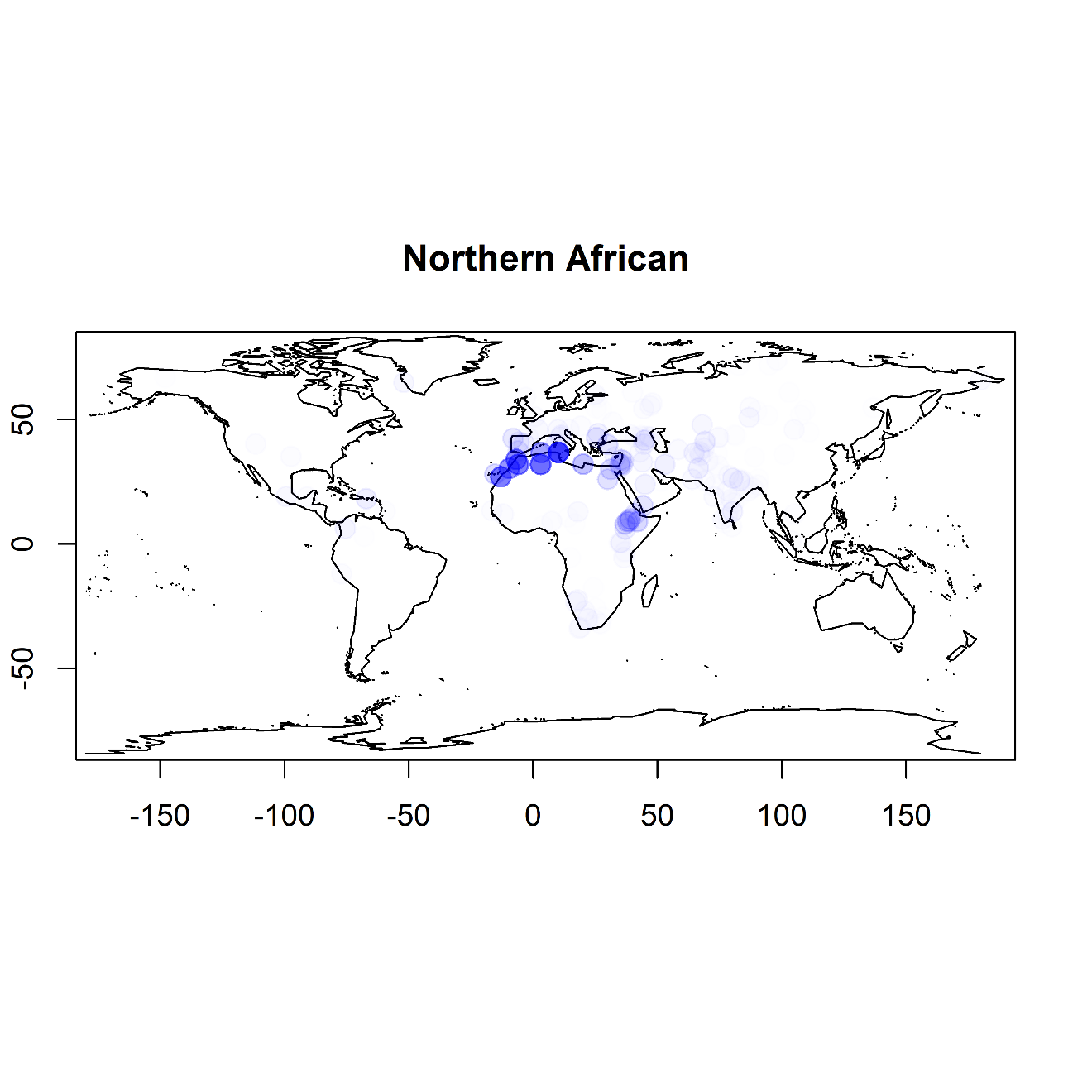


Figure S2. (H) Present-day geographic distribution of Northern African ancestry. The intensity of blue is directly proportional to the denoised and renormalized sample mean.


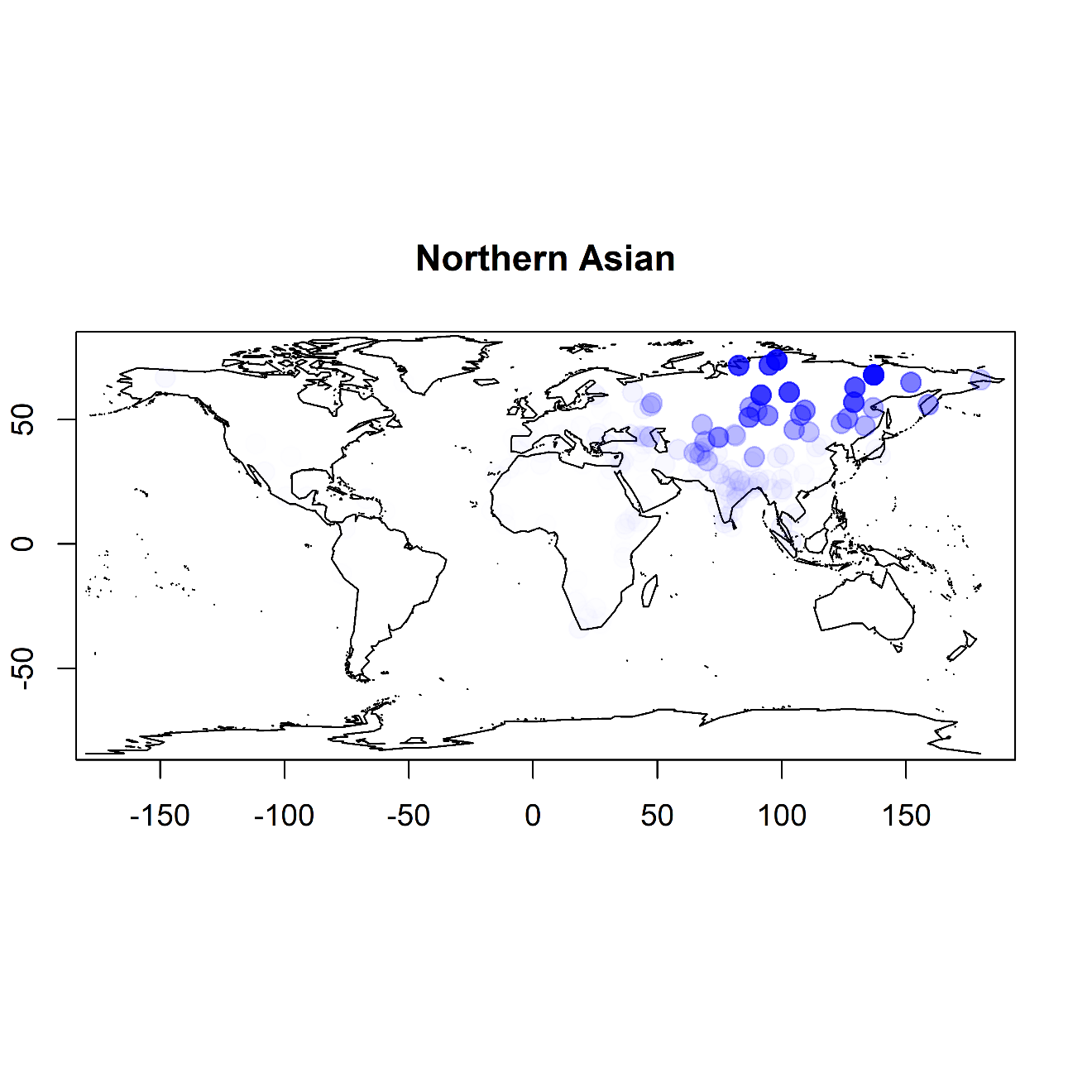


Figure S2. (I) Present-day geographic distribution of Northern Asian ancestry. The intensity of blue is directly proportional to the denoised and renormalized sample mean.


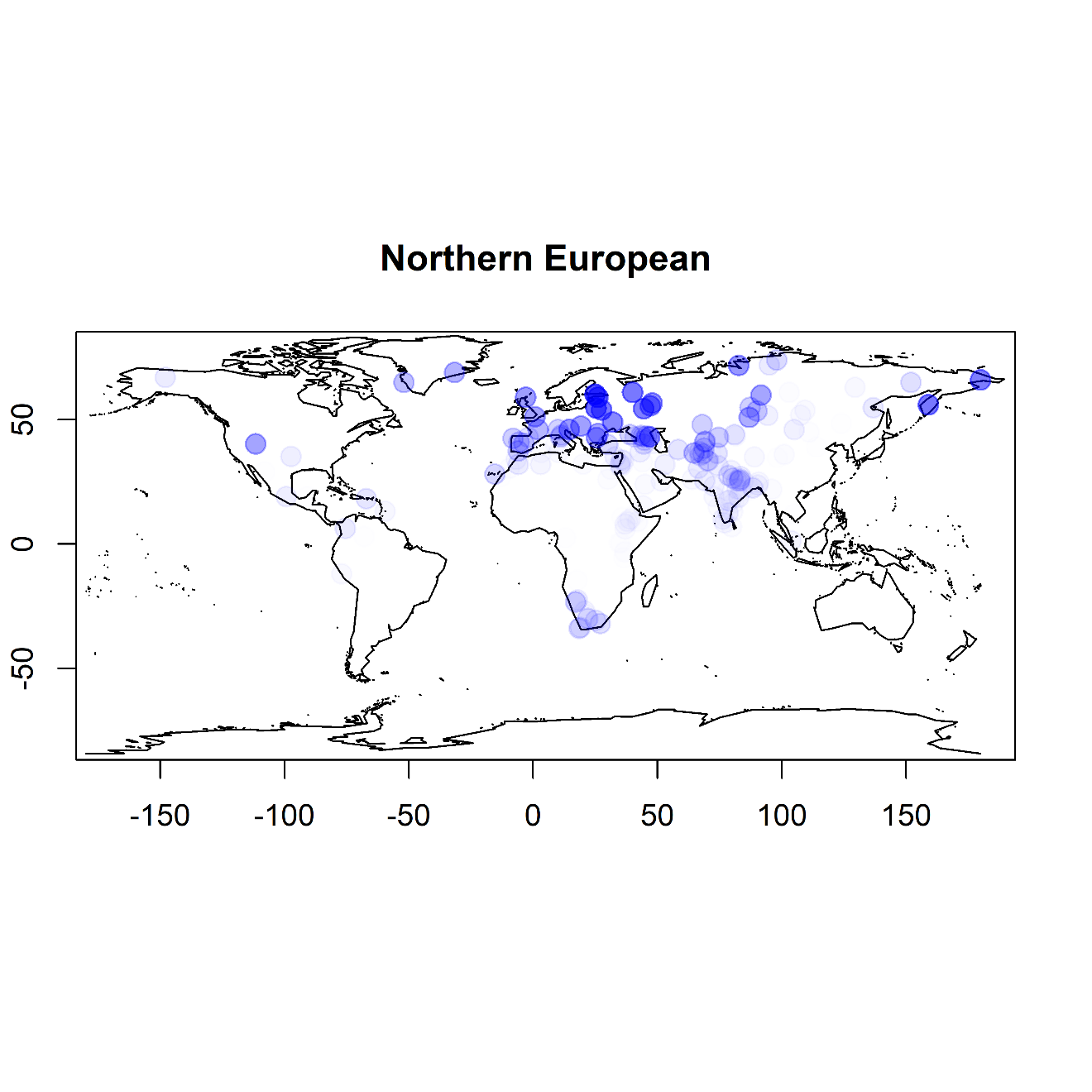


Figure S2. (J) Present-day geographic distribution of Northern European ancestry. The intensity of blue is directly proportional to the denoised and renormalized sample mean.


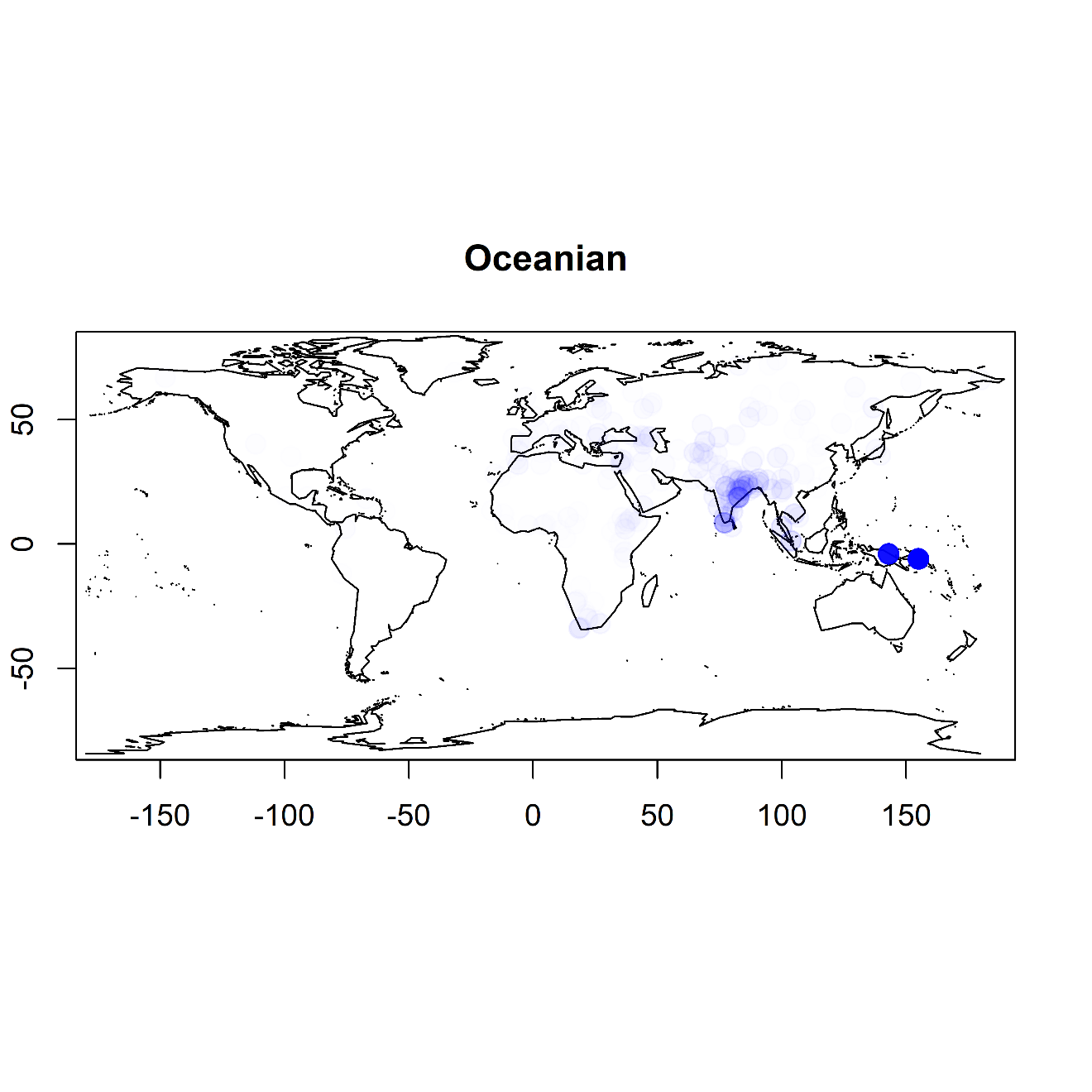


Figure S2. (K) Present-day geographic distribution of Oceanian ancestry. The intensity of blue is directly proportional to the denoised and renormalized sample mean.


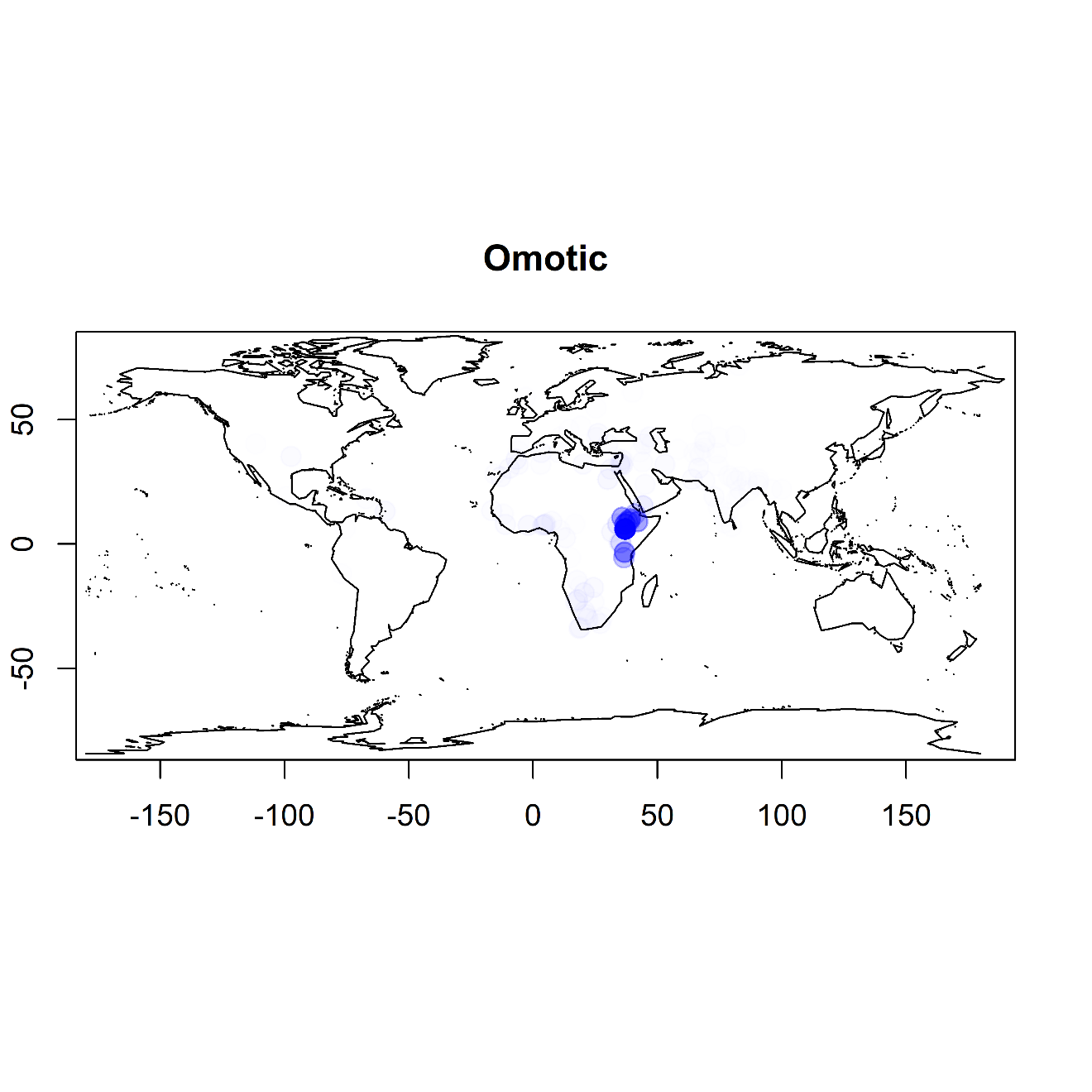


Figure S2. (L) Present-day geographic distribution of Omotic ancestry. The intensity of blue is directly proportional to the denoised and renormalized sample mean.


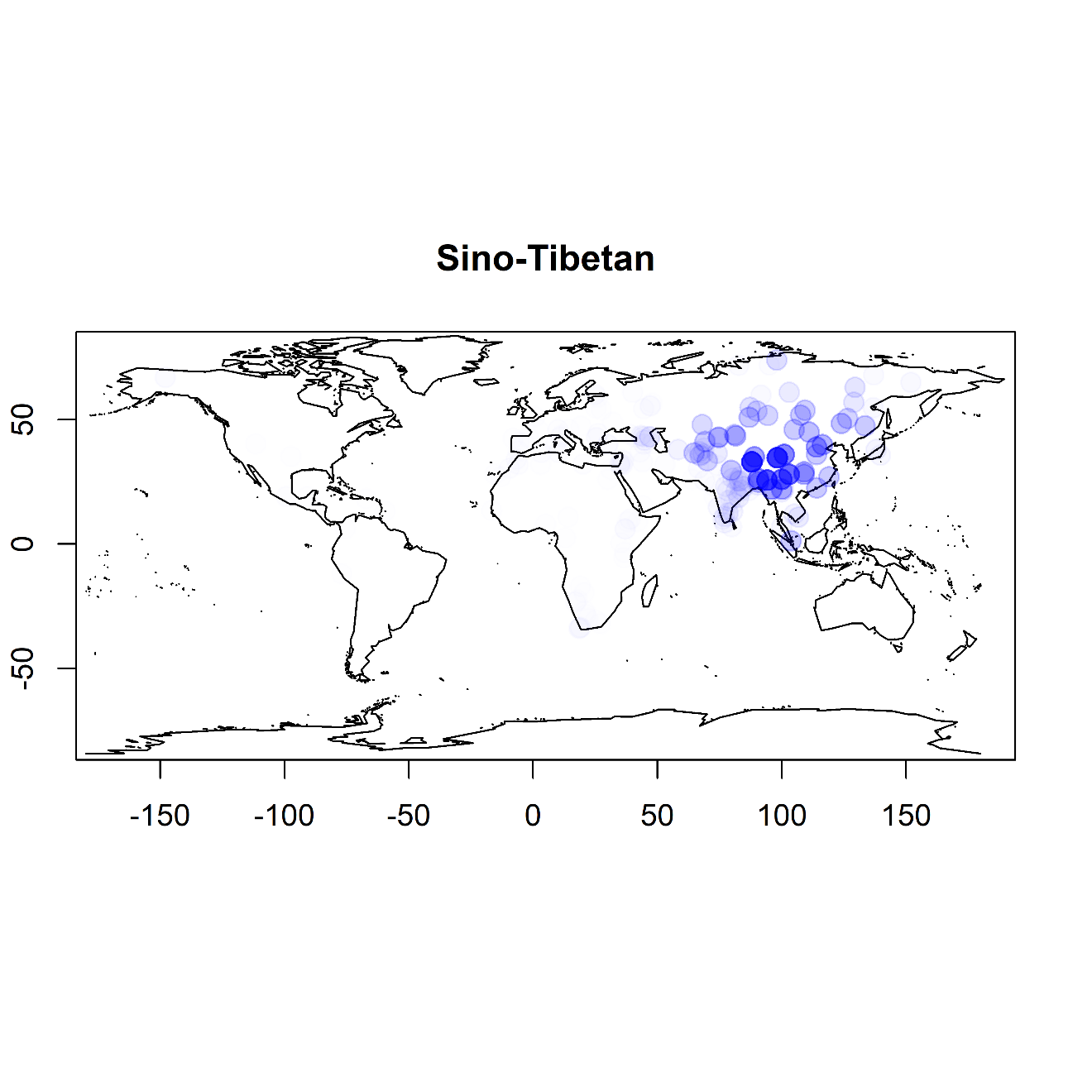


Figure S2. (M) Present-day geographic distribution of Sino-Tibetan ancestry. The intensity of blue is directly proportional to the denoised and renormalized sample mean.


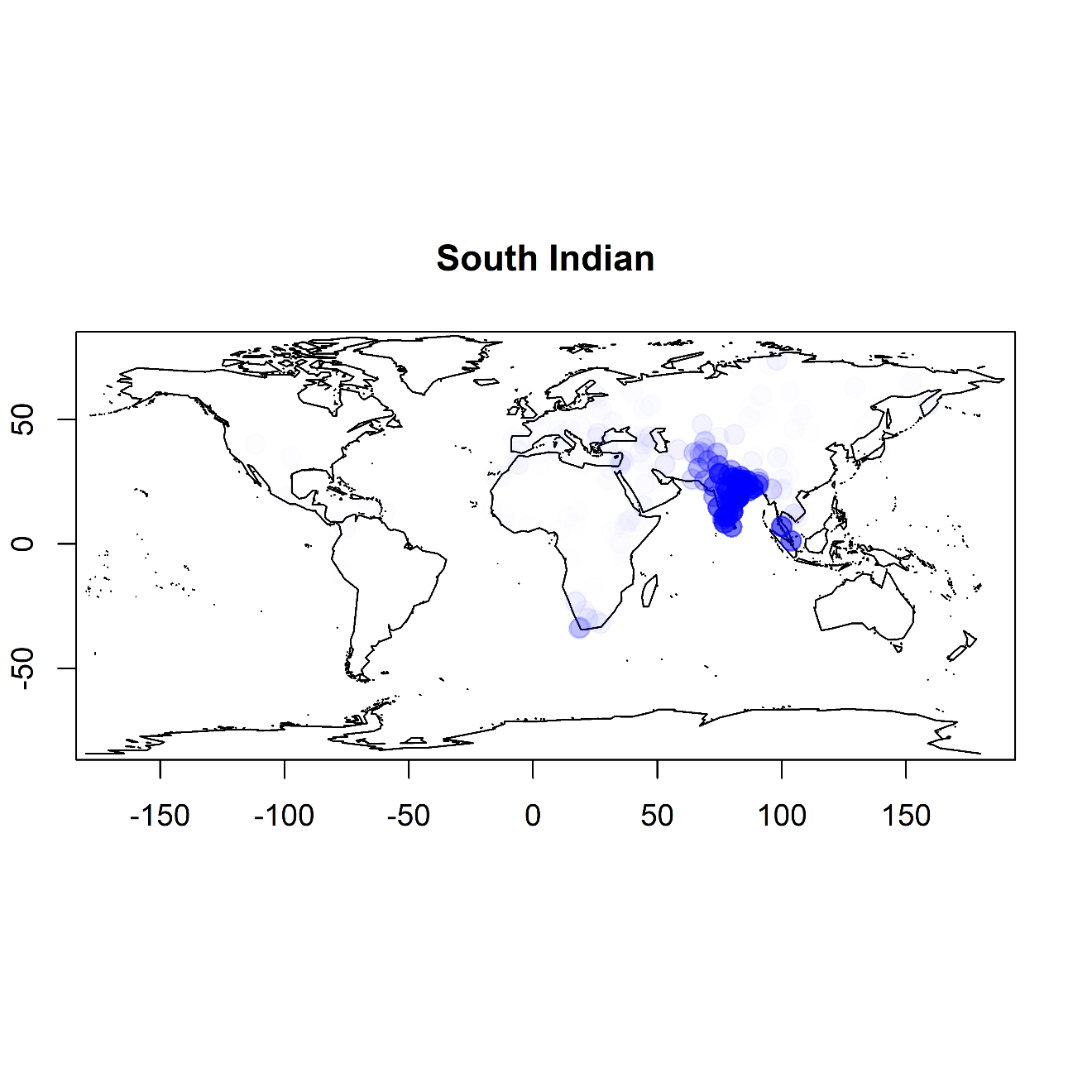


Figure S2. (N) Present-day geographic distribution of South Indian ancestry. The intensity of blue is directly proportional to the denoised and renormalized sample mean.


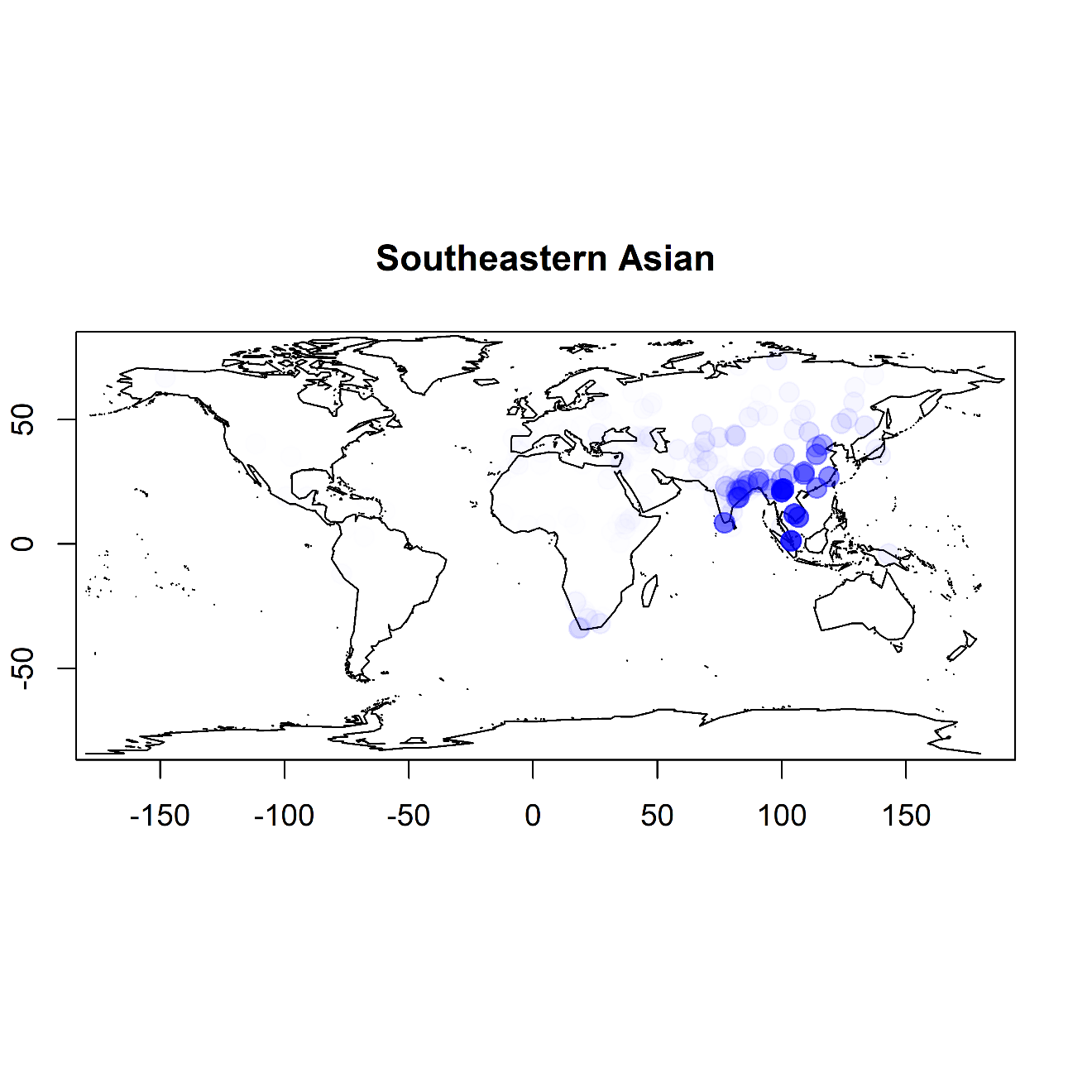


Figure S2. (O) Present-day geographic distribution of Southeastern Asian ancestry. The intensity of blue is directly proportional to the denoised and renormalized sample mean.


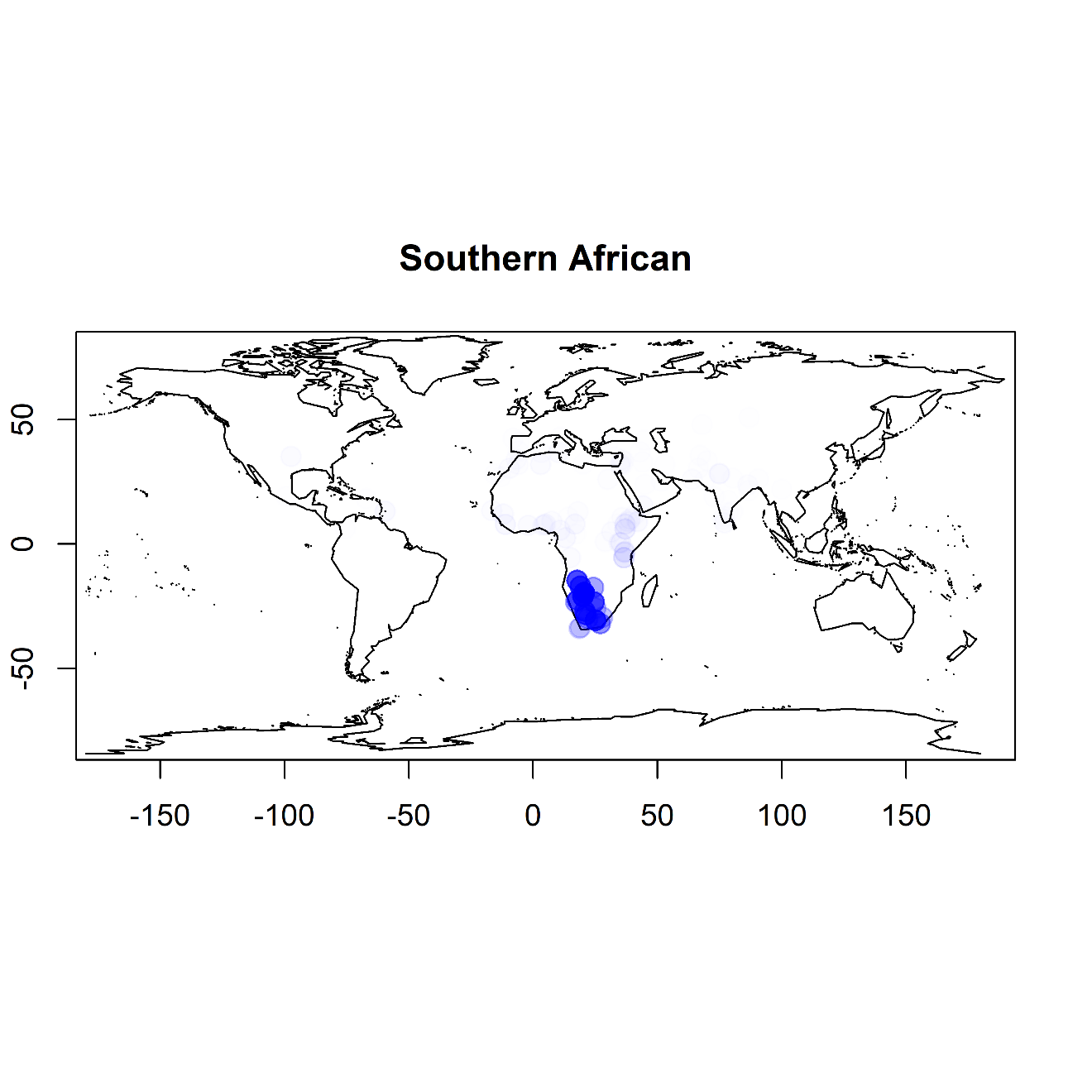


Figure S2. (P) Present-day geographic distribution of Southern African ancestry. The intensity of blue is directly proportional to the denoised and renormalized sample mean.


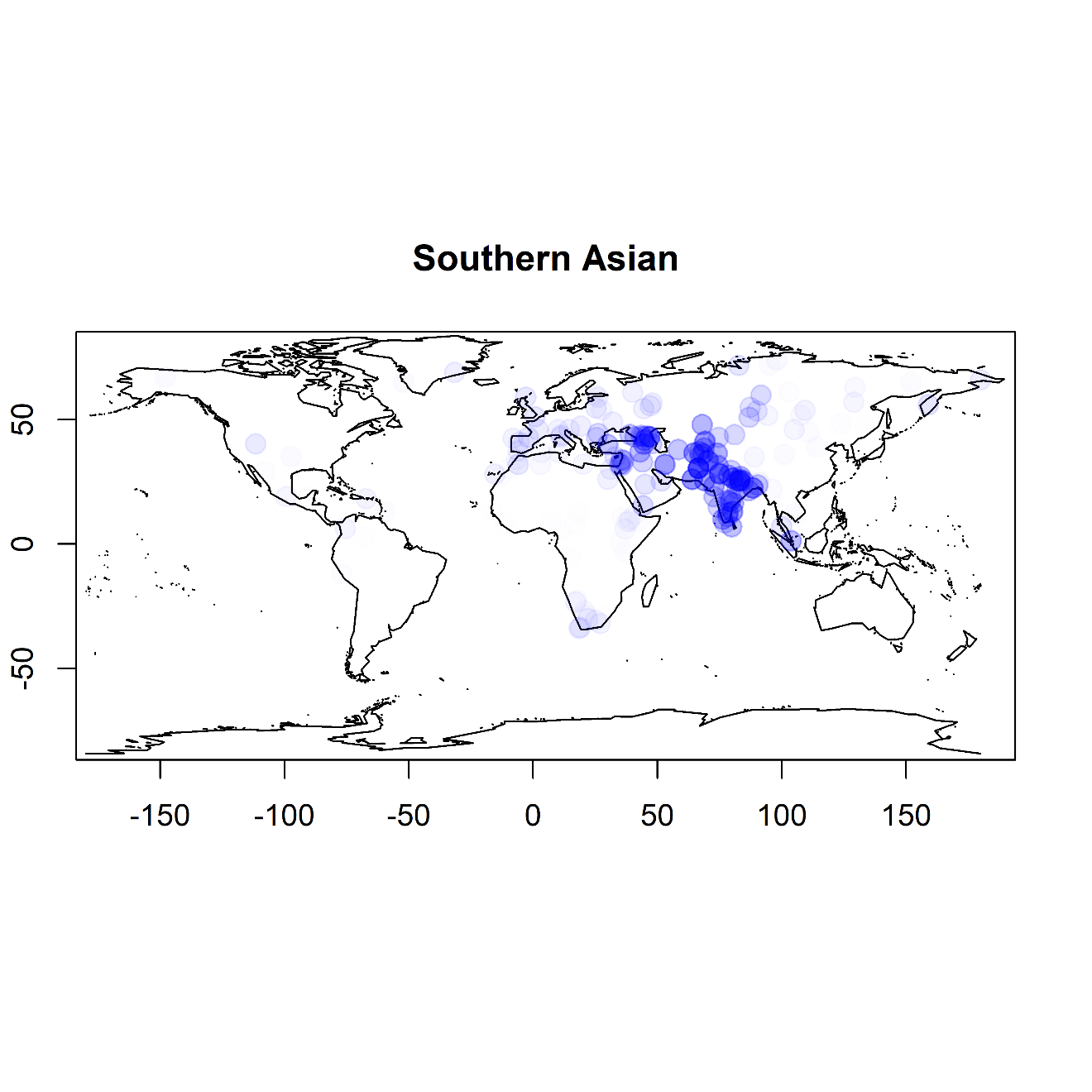


Figure S2. (Q) Present-day geographic distribution of Southern Asian ancestry. The intensity of blue is directly proportional to the denoised and renormalized sample mean.


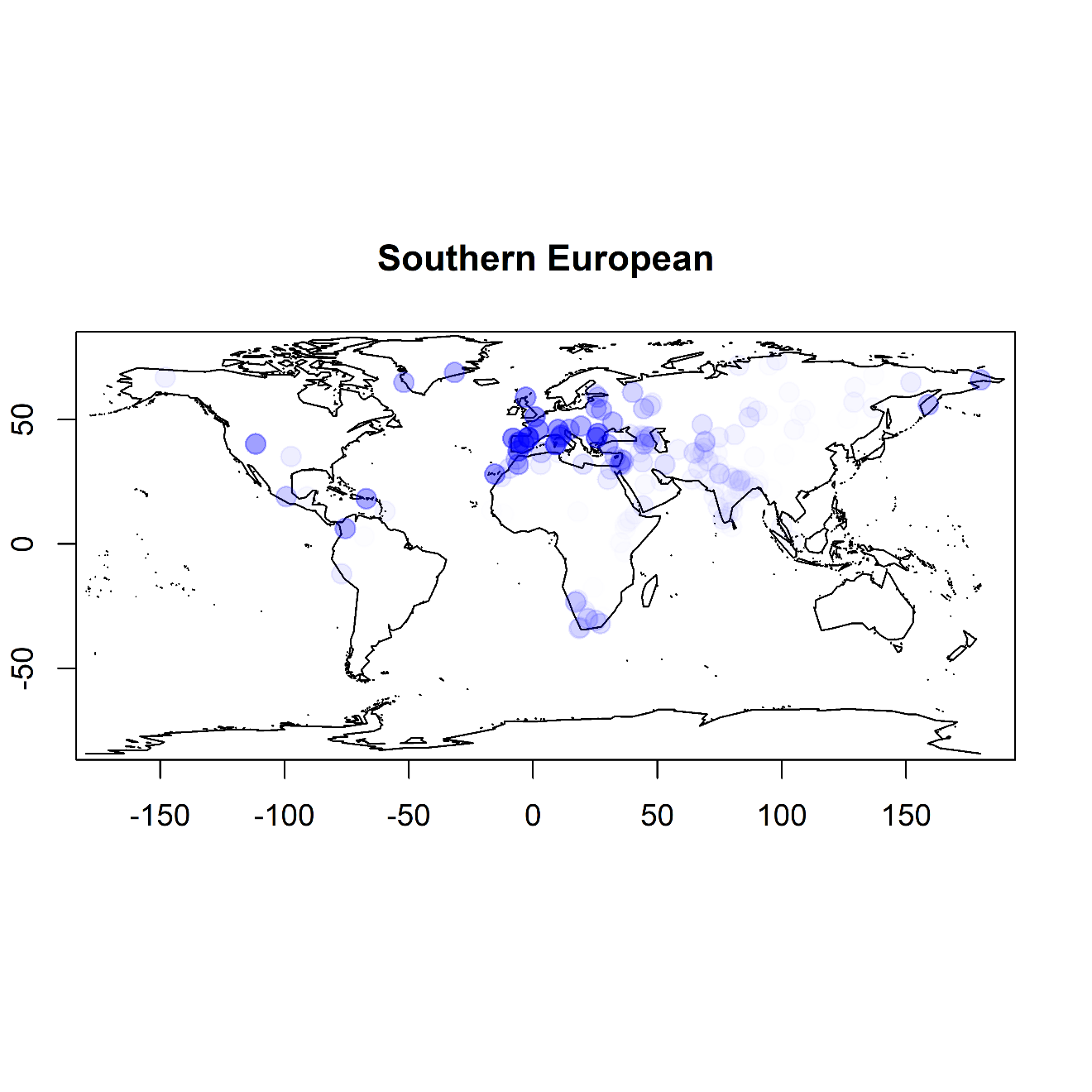


Figure S2. (R) Present-day geographic distribution of Southern European ancestry. The intensity of blue is directly proportional to the denoised and renormalized sample mean.


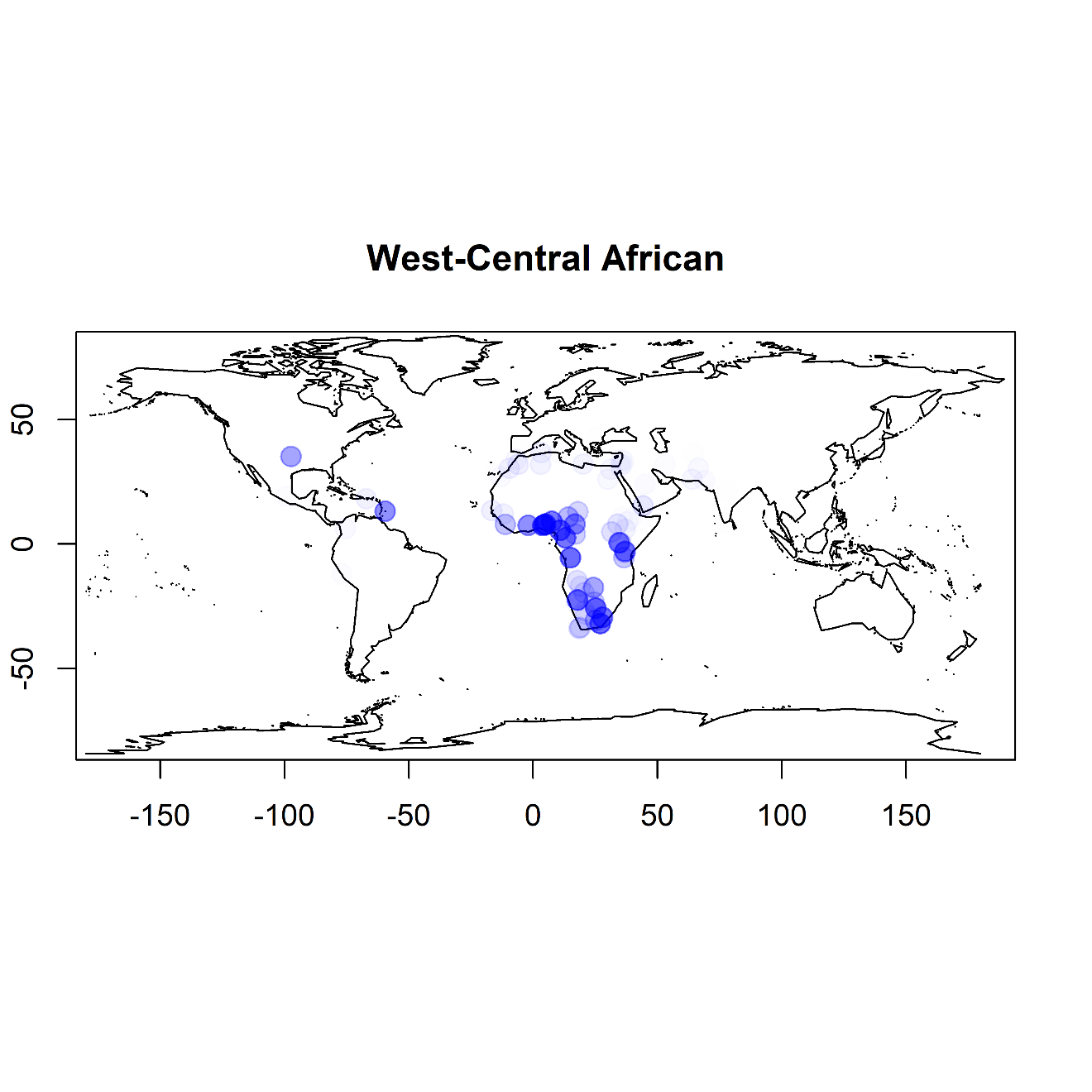


Figure S2. (S) Present-day geographic distribution of West-Central African ancestry. The intensity of blue is directly proportional to the denoised and renormalized sample mean.


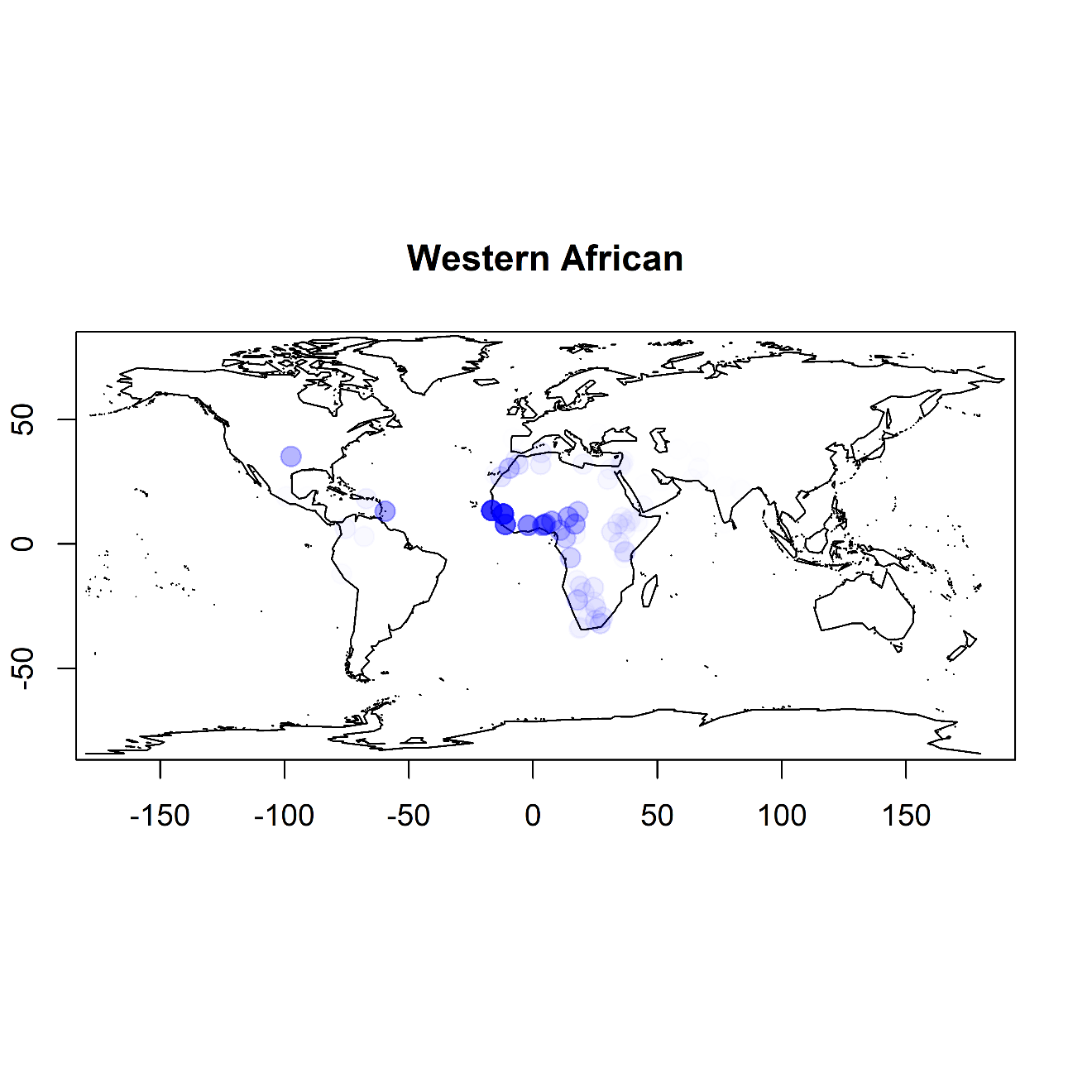


Figure S2. (T) Present-day geographic distribution of Western African ancestry. The intensity of blue is directly proportional to the denoised and renormalized sample mean.


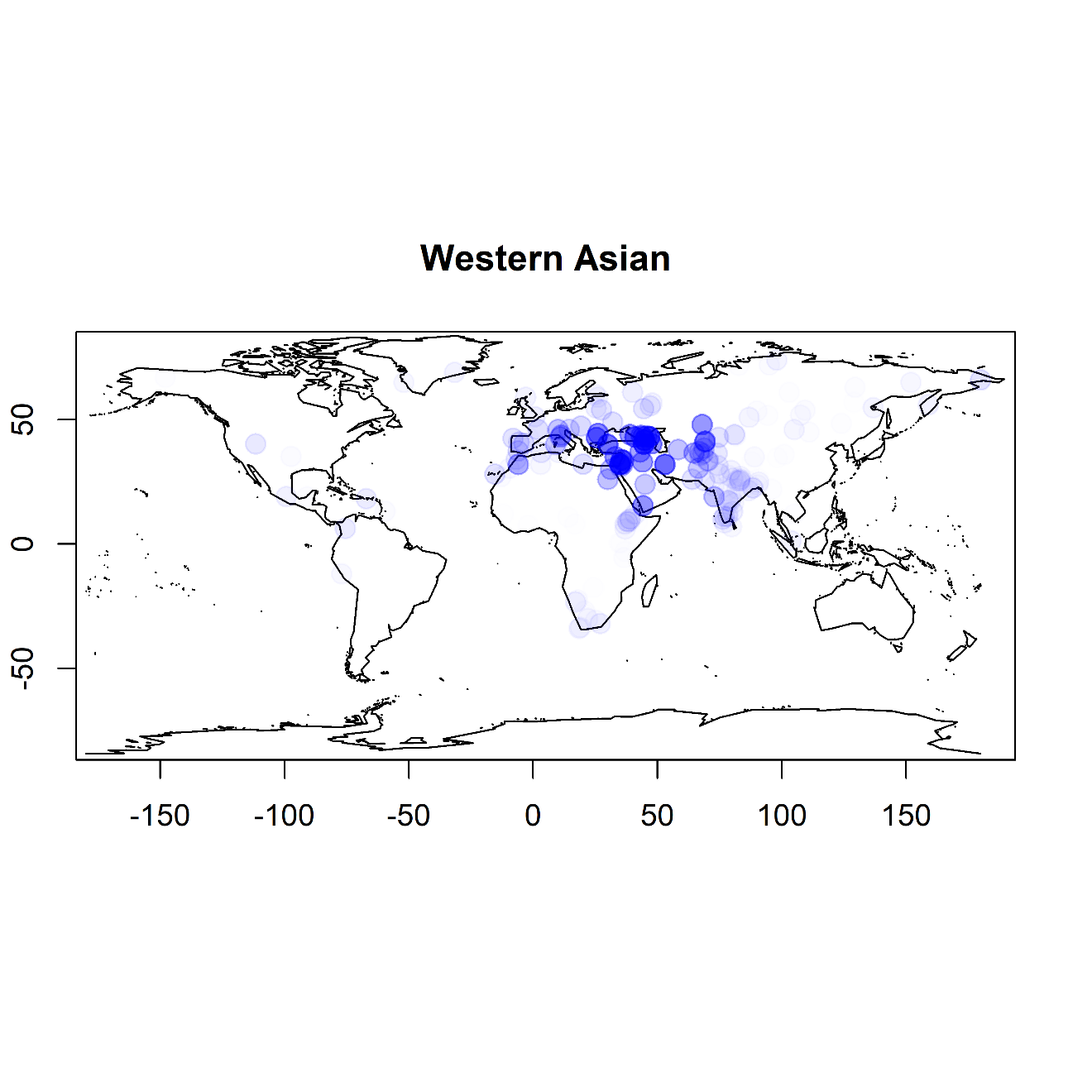


Figure S2. (U) Present-day geographic distribution of Western Asian ancestry. The intensity of blue is directly proportional to the denoised and renormalized sample mean.
